# Controlling Chemical Dynamics of Molecular Assemblies through Nanoconfinement: *o*‑Nitrosocumene@Pd Nanocage

**DOI:** 10.1021/acs.joc.6c00239

**Published:** 2026-07-07

**Authors:** Anu Pradeep, Cory H. Rogers, Radek Marek, Silas C. Blackstock, Vaidhyanathan Ramamurthy

**Affiliations:** † Department of Chemistry, 5452 University of Miami, Coral Gables, Florida 33146, United States; ‡ Department of Chemistry and Biochemistry, 8059 The University of Alabama, Tuscaloosa, Alabama 35487-0336, United States; § Department of Chemistry, Faculty of Science, Masaryk University, Kamenice 5, Brno 62500, Czechia; ∥ National Center for Biomolecular Research, Faculty of Science, Masaryk University, Kamenice 5, Brno 62500, Czechia

## Abstract

The change in chemical dynamics of confined *ortho*-nitrosocumene (*o*-NC), as observed by ^1^H NMR spectroscopy, illustrates the power of nanoconfinement to alter reaction mechanisms from those observed in isotropic solutions where molecules have more freedom. When *o*-NC molecules that exist as a dynamic mixture of monomer (M), covalent dimers (D), and supramolecular aggregates (A) are provided a choice of being free in water or confined within an organometallic cage (Pd nanocage, PdNC), they prefer the latter. The confinement significantly alters the molecular distribution such that A no longer exists, and new equilibria among M and *Z*/*E* isomers of D are established within the nanocage and with components in the aqueous exterior. The change in equilibrium distribution of guest components demonstrates how the *o*-NC molecular forms of A, M, and D depend not only on concentration and temperature but also on the microenvironment surrounding the molecules. Temperature-dependent NMR and exchange spectroscopy (EXSY) with various mixing times are used to explore the M ⇄ D equilibrium and the D_*Z*_ ⇄ D_*E*_ interchange within the PdNC host. Nanoconfinement in the cage influences the dynamic interplay of dissociation, rotation, translation, and recombination events in these processes. Strikingly, the previously unreported D_*Z*_-to-D_*E*_ exchange bypassing splitting to free M is enabled by entropy control in the nanoreactor.

## Graphical abstract

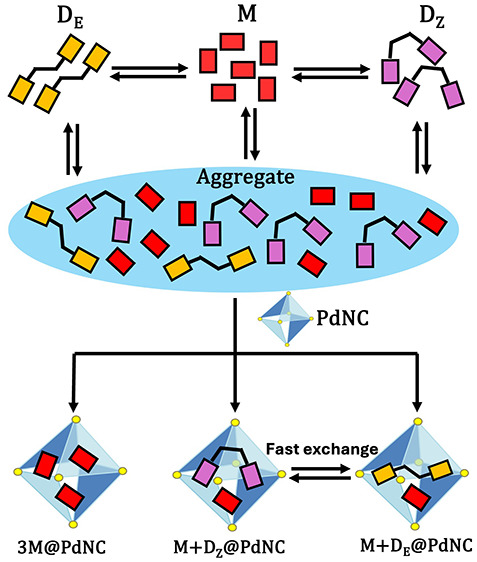

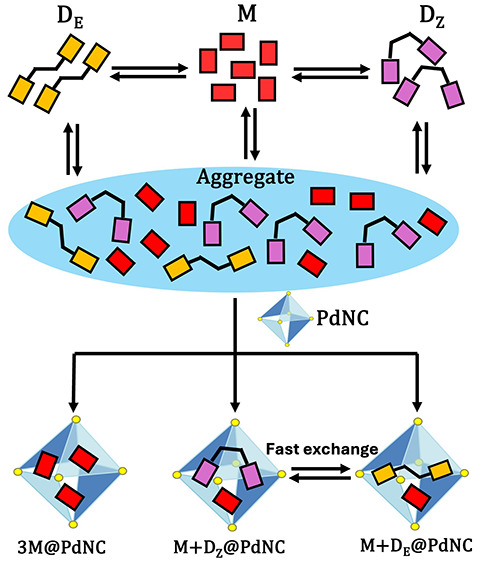

## Introduction

This article presents the results of our studies on the dynamic behavior of *o*-nitrosocumene (*o*-NC) in water in the presence of a well-characterized supramolecular host Pd^12+^ nanocage (PdNC, Scheme 1, left). Compared to many other organic chromophores, the nitroso function (−N=O) is less well investigated^1,2^ but recently its value in molecular, polymer, and materials synthesis has attracted attention.^3–9^ In addition to experimental, computational approaches have been attempted to understand the structure and reactivity of molecules with nitroso functionality.^10–15^ The well-separated excited states (S_2_ and S_1_) of nitrosoarenes that attracted the attention of spectroscopists early on are fraught with problems due to their propensity to establish an equilibrium with their dimers in solution, leading to spurious results.^16–18^ Even though not all nitrosoarenes dimerize in solution at room temperature, many of them have a low barrier leading to a dynamic monomer ⇄ dimer (M ⇄ D) equilibrium in solution.^4,11,12,19–23^ Such equilibria in solution and in the crystalline state have been the subject of several investigations.^24–30^ In this study, we have explored the possibility of altering the M–D equilibrium of *o*-NC by confining it in a restricted space. We have recently shown that such an approach allows one to selectively trap *o*-NC M within organic hosts, such as cyclodextrin (CD), cucurbituril (CB), and octa acid (OA) in water.^31,32^ This prompted us to explore a larger host cavity, namely, Fujita’s Pd^12+^ nanocage ([M_6_L_4_]^12+^, PdNC),^33,34^ which possesses a larger internal space and multiple openings and affords a new venue to explore *o*-NC ensemble binding and potential intracage reactions and dynamics of this guest (Scheme 1).

**Scheme 1:**
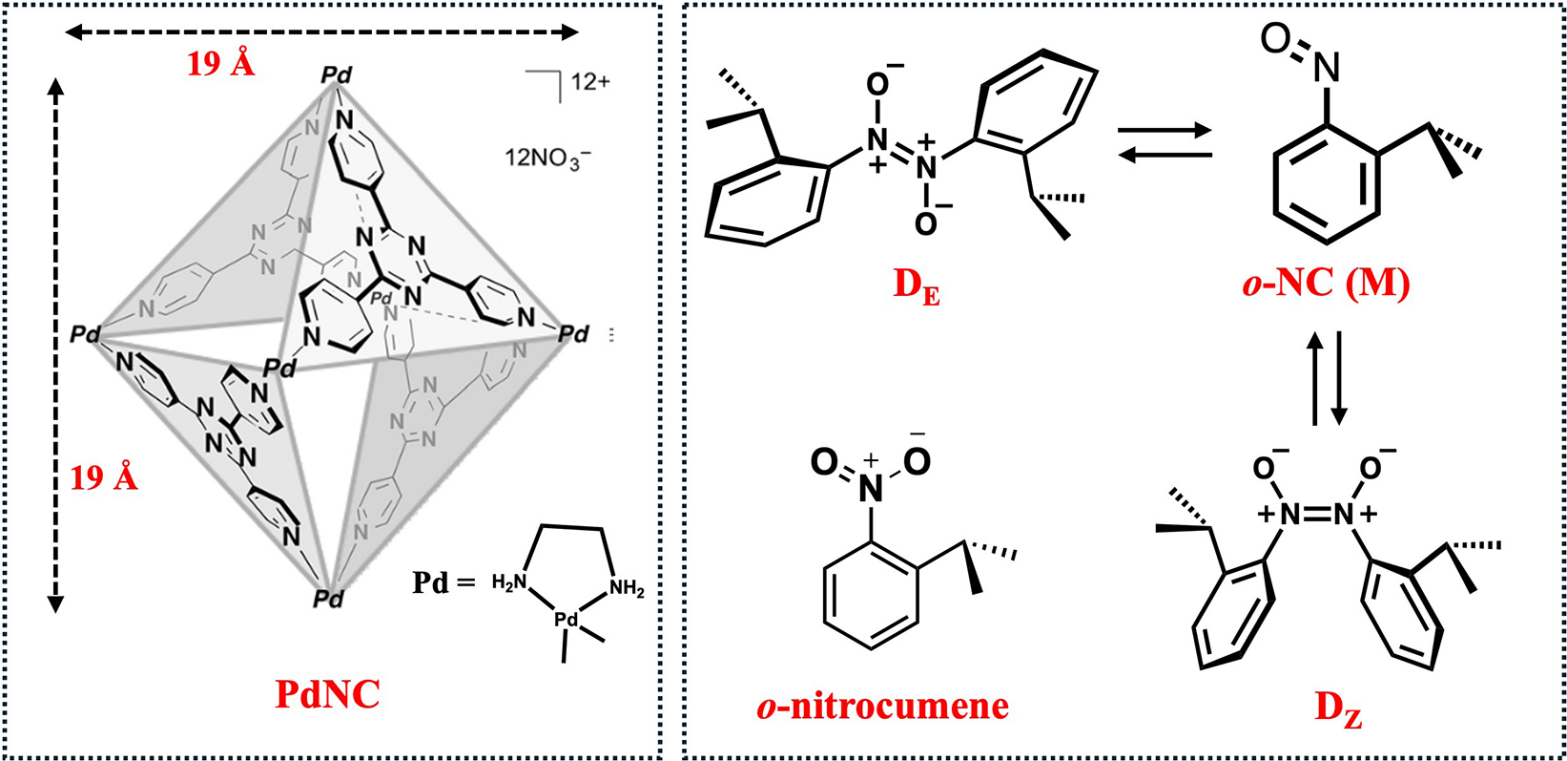
Structures of Supramolecular PdNC Host (left) and *o*-NC and *o*-nitrocumene Guest molecules (right) Employed in This Study

The motivation to pursue this strategy to alter the M–D equilibrium of *o*-NC came from the established control over thermal and photoreactions in host–guest supramolecular assemblies that range from flexible and dynamic micelle enclosures to more rigid and time-independent crystal environments.^35–37^ In these restricted environments, factors that do not play a role in solution, such as "free space", are likely to be important. A qualitative model based on size, shape, and rigidity (soft/hard) of the reaction cavity, the extent and location of free space within the cavity, and weak supramolecular interactions between the guest and the host allow some predictions about reactions occurring in this medium.^38,39^ Given that each host system in terms of internal structure and openings is unique, we anticipated that the behavior of *o*-NC would be different within PdNC compared to that within CD, CB, and OA.^31^

Recently, we showed via ^1^H NMR studies that the dynamic guest of this study *o*-NC exists in six distinct and interconverting forms in water (Figure 1a), all with resolved ^1^H NMR signals (Figure 1b).^23,40^ The structures include monomer (M), *Z*- and *E*-azodioxide dimers (D_*Z*_, D_*E*_), and an aggregate (A) of these containing A_M_, A_DZ_, and A_DE_, all with unique NMR signals. Figure 1 shows ^1^H NMR spectra of *o*-NC at different concentrations; the signals for *i*-Pr methyl groups are indicated. These signals for M, D, and A are color-coded. At a low concentration (0.2 mM), there is only M. At a slightly higher concentration (0.5 mM), both M and D are present. Compared to organic solvents, water drives the equilibrium more toward D.^23^ We attribute the enhanced D formation in water to the hydrophobic effect, preferred hydrogen bonding between D and solvent molecules, and to the higher polarity of water than organic solvents.^23^ At higher concentrations in water (>0.5 mM), above the solubility limit of M and D, aggregates also appear. As observed in the NMR spectrum, at 2.0 mM, the aggregate phase contains both M and D structures. The distinct NMR signals for free M and D as well as for aggregated A_M_ and A_D_ allowed us to employ *o*-NC to probe the influence of PdNC on the dynamic behavior of nitrosoarenes.

**Figure 1:**
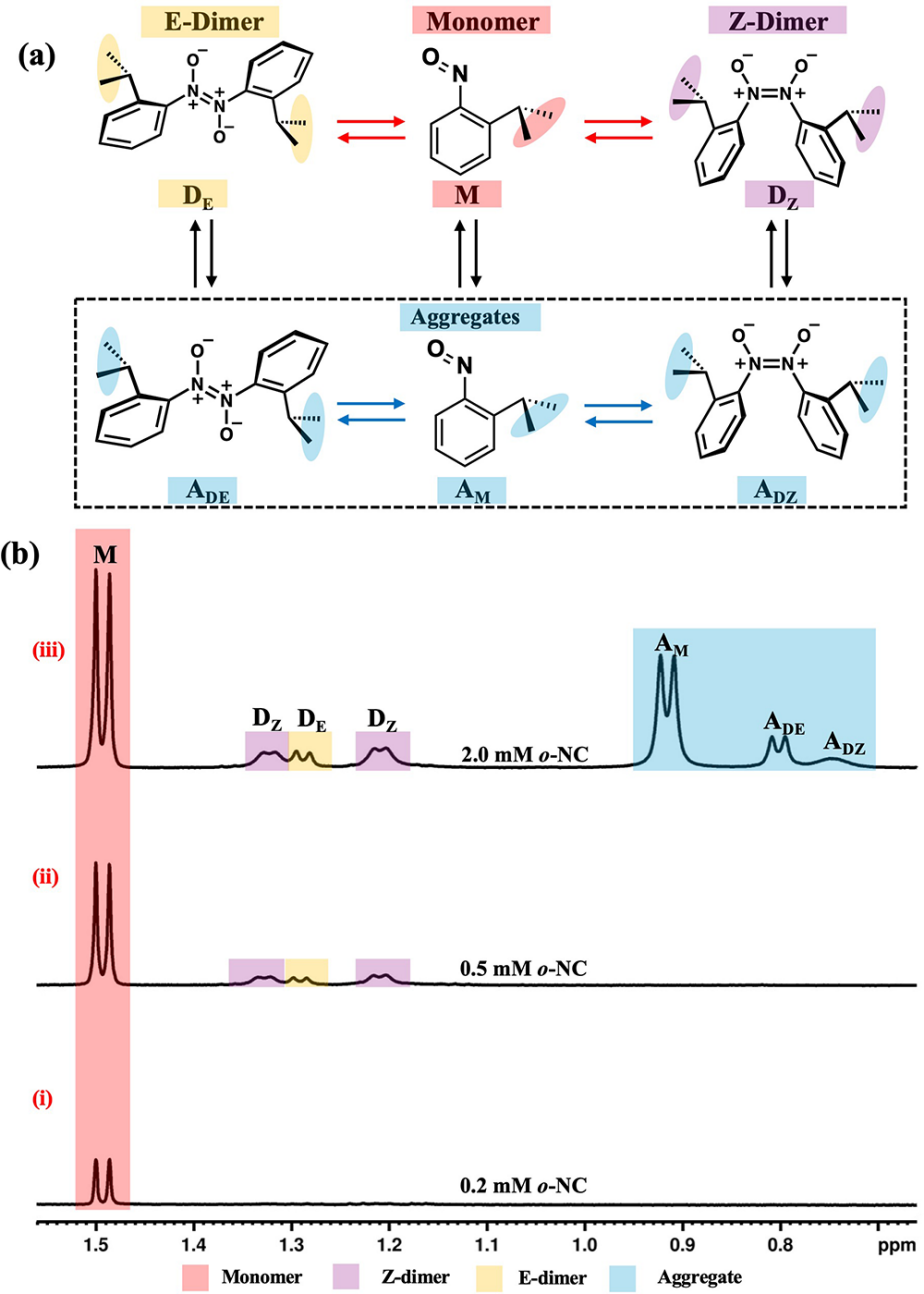
(a) Schematic of *o*-NC assemblies in aqueous solution. (b) ^1^H NMR spectra (500 MHz, D_2_O, 25 °C) of *o*-NC methyl signals in water at various concentrations demonstrating the coexistence of monomeric (M), dimeric (D_*Z*_, D_*E*_), and aggregate (A) forms.

It is noteworthy that both D_*E*_ and D_*Z*_ structures have conformations with cumyl groups twisted significantly out of the azodioxide plane, as supported by DFT calculations (58° twist for D_*E*_, 63° twist for D_*Z*_) (Figure S26) and by the X-ray structure of *o*-NC D_*Z*_ solid (74° twist). The twisted cumyl rings of these *o*-NC dimer structures possess diastereotopic methyl groups, which are well resolved by NMR for D_*Z*_ (shows two methyl NMR signals) but not for D_*E*_ (shows one averaged methyl NMR signal) in D_2_O solution. Rotation about both C,N (cumyl) bonds averages the methyl environments in these dimers and is apparently slow on the NMR time scale for the more sterically congested D_*Z*_ structure, but fast on the NMR time scale for the less sterically congested D_*E*_ structure, leading to a single, dynamically averaged methyl signal of the *i*-Pr group for D_*E*_ in D_2_O (Scheme 1 and Figure S26).^23^ Interestingly, single methyl NMR signals for isopropyl groups of *o*-NC M, D_*E*_, and D_*Z*_ are observed for all three structures in the aggregated environment, although the D_*Z*_ methyl signal is broad, probably reflecting a slowed, but not yet frozen-out, cumyl rotation for D_*Z*_ in the aggregate phase at ambient temperature. The slowed cumyl rotation for D_*Z*_ in D_2_O compared to that in the aggregate may reflect a more compacted D_*Z*_ structure in water with hydrogen-bonded solvent at the oxide oxygen atoms, which perhaps hinders the cumyl rotation process, leading to well-resolved diastereotopic methyl signals in the NMR spectrum at room temperature. Chemical exchange, observed previously^23^ by EXSY NMR in D_2_O between M and D (red arrows of Figure 1), between A_M_ and A_D_ (blue arrows of Figure 1), and between dissolved and aggregate *o*-NC forms (black arrows of Figure 1), has allowed the complete NMR signal assignment of the six forms of *o*-NC in D_2_O as depicted for methyl signals shown in Figure 1.

Previous reports have shown that multiple molecules (e.g., adamantane derivatives and *o*-carborane) can be included within PdNCs.^41^ Also, reports of [2 + 2] photodimerization of two different olefins and the [4 + 2] Diels–Alder reactions of a few diene–dienophile pairs within PdNC suggested that bimolecular reactions are feasible in this nanoreactor.^42,43^ Thus, exploration of PdNC host chemistry with *o*-NC offers the possibility to include more than one nitrosoarene molecule and potentially explore the *o*-NC dynamic covalent equilibrium (2 M ⇄ D) within the enclosed space. Based on the results within the OA capsule, where 2 M bind but do not combine to D, we are aware that inclusion of two molecules in a small space alone does not guarantee dimerization.^31^ In this study, we have investigated the feasibility of manipulating the *o*-NC assembly distribution in water by selectively trapping one or more *o*-NC forms within a PdNC host. The results presented here highlight the value of "confined space and its character" as a tool for influencing complex chemical equilibria and their dynamics by introducing new supramolecular reaction components.

## Results and Discussion

### Host–Guest Complexation—General Observations

For the current study, a 2.0 mM aqueous translucent suspension of *o*-NC that exists as a mixture of approximately 16% M, 14% D (*Z* and *E* isomers), and 70% A (Figure 1) was used. Under this condition, only ∼86% of the total *o*-NC could be detected by ^1^H NMR. The remainder of the undetected *o*-NC exists as large aggregates that could be observed only by dynamic light scattering. Upon the addition of PdNC, this solution becomes transparent and 100% of *o*-NC becomes visible to NMR as M and D. At this stage, no A remains. It is important to note that aggregation occurs only beyond the solubility limit of *o*-NC in water (0.5 mM). In the presence of PdNC, after M and D enter the cage, the solution allows more A to dissociate into dissolved (aqueous) M and D. In the presence of sufficient amounts of PdNC, all A dissociates, and the solution becomes transparent. As discussed below, addition of PdNC results in sequestration of *o*-NC as M and D within the cage, shifting the *o*-NC equilibria in D_2_O toward the soluble guest@PdNC. Analysis of host–guest (H-G) complexation and guest reaction dynamics has been monitored primarily by the changes in the ^1^H NMR signals of the *i*-Pr group of the *o*-NC guests, which are well resolved in all environments. Changes in other signals are also noted and informative. Due to the involvement of multiple guest *o*-NC equilibria in water, which accompany PdNC binding, isothermal titration calorimetry (ITC) measurements could not be used to evaluate the binding constants of M and D to PdNC. Despite this limitation, we have been able to make reasonably definite conclusions about *o*-NC structural forms and interconversions, which are unique to PdNC binding and different from those noted earlier for *o*-NC complexes with OA, CB, and CD.^31^

### Determination of Host–Guest Ratio—Nondimerizing *o*-Nitrocumene as the Model Guest

Initially, to determine how many monomeric *o*-NC molecules might fit within the PdNC cavity, we chose *o*-nitrocumene, a molecule of similar size to *o*-NC, as a nondimerizable *o*-NC model guest. NMR titration spectra displaying changes in the guest and host signals (Figures S2 and S3) suggest that ∼1.9 mM of PdNC host is required to fully include 6.0 mM of *o*-nitrocumene guest. As shown in Figure S2, 6.0 mM of *o*-nitrocumene exists in D_2_O as a dissolved monomer species (*i*-Pr Me signal of *o*-nitrocumene, δ 1.25 ppm) and as an aggregate species (*i*-Pr Me, δ 0.82 ppm). As the host is added, a new bound *o*-nitrocumene *i*-Pr Me signal is observed, under fast exchange with dissolved monomer *o*-nitrocumene. The integration of this signal relative to the host H_c_ signal is 3­(*o*-nitrocumene)@PdNC (Figure S2ii–v) until the H:G ratio exceeds 3:1 (Figure S2vi). Thus, with excess *o*-nitrocumene guest, three guest molecules bind to one PdNC. Further confirmation of this 3G:1H binding stoichiometry is observed in the host NMR signal H_a_ (Figure S3v,vi), which shows a clear break only after the H:G ratio exceeds 1:3, to give a host, which is not fully occupied by guest. A point to note is that when more host is added beyond the 1:3 H:G level, making more cages available in solution, the host signal shifts toward free PdNC (compare traces (vi) to (ix) with (x) in Figure S3). This suggests that the *o*-nitrocumene@PdNC complex is dynamic, and the guest location exchanges between the hydrophobic PdNC interior and hydrophilic aqueous exterior. The dynamic nature is evident when the guest M signal is followed in Figure S2. The M signal continuously shifts with the addition of PdNC. Had the H-G complex been static on the NMR time scale, there would have been two signals, one for bound and one for free guest, the ratio changing with the addition of the host. The PdNC cavity space for three *o*-nitrocumene guests raises an interesting question: if three *o*-NC molecules were included in the PdNC cavity, would they establish a M ⇄ D equilibrium like that in solution?

### Complexation of *o*-NC with PdNC—Addition of Host to Guest

Following the above experiments with *o*-nitrocumene, the ^1^H NMR titration spectra of *o*-NC and PdNC were recorded in two modes: (a) gradual addition of PdNC to an *o*-NC solution (Figure 2 and Figures S4 and S5) and (b) gradual addition of *o*-NC to a PdNC solution (Figure 3 and Figure S6). In Figure 2, guest NMR signals show a gradual evolution to more shielded positions as PdNC is added, up to a point of a H:G ratio of 1:3, consistent with the anticipated capture of three *o-*NC guests by a single PdNC host. Notably, the bound guest *i*-Pr signals appear as multiple resonances, which are assigned as bound M, D_*Z*_, or D_*E*_ *o-*NC structures (assignments below), indicating dimer structures are observed within the PdNC host, the first instance of a host-bound nitrosobenzene dimer. With the addition of host PdNC beyond the 1:3 H:G ratio, the guest signals do not change much (Figure 2), which we interpret to mean that most guest molecules stay as a group of three in the host cage rather than distributing themselves among more cages having fewer guests. At this stage, we are unsure of the exact reason for this preference, but considering that PdNC includes only three molecules of similarly sized *o*-nitrocumene, we believe the cavity size is a factor. We cannot rule out any interaction between the Pd atoms of the nanocage and the guest molecule. When the concentration of the host exceeds 1:3 H:G, one would expect the emergence of signals for free host. The fact that it is not seen in Figure S4 suggests that there is fast exchange between the complexed and free hosts, the guest being present within the host most of the time as M and D, with the binding being dynamic in nature.

**Figure 2:**
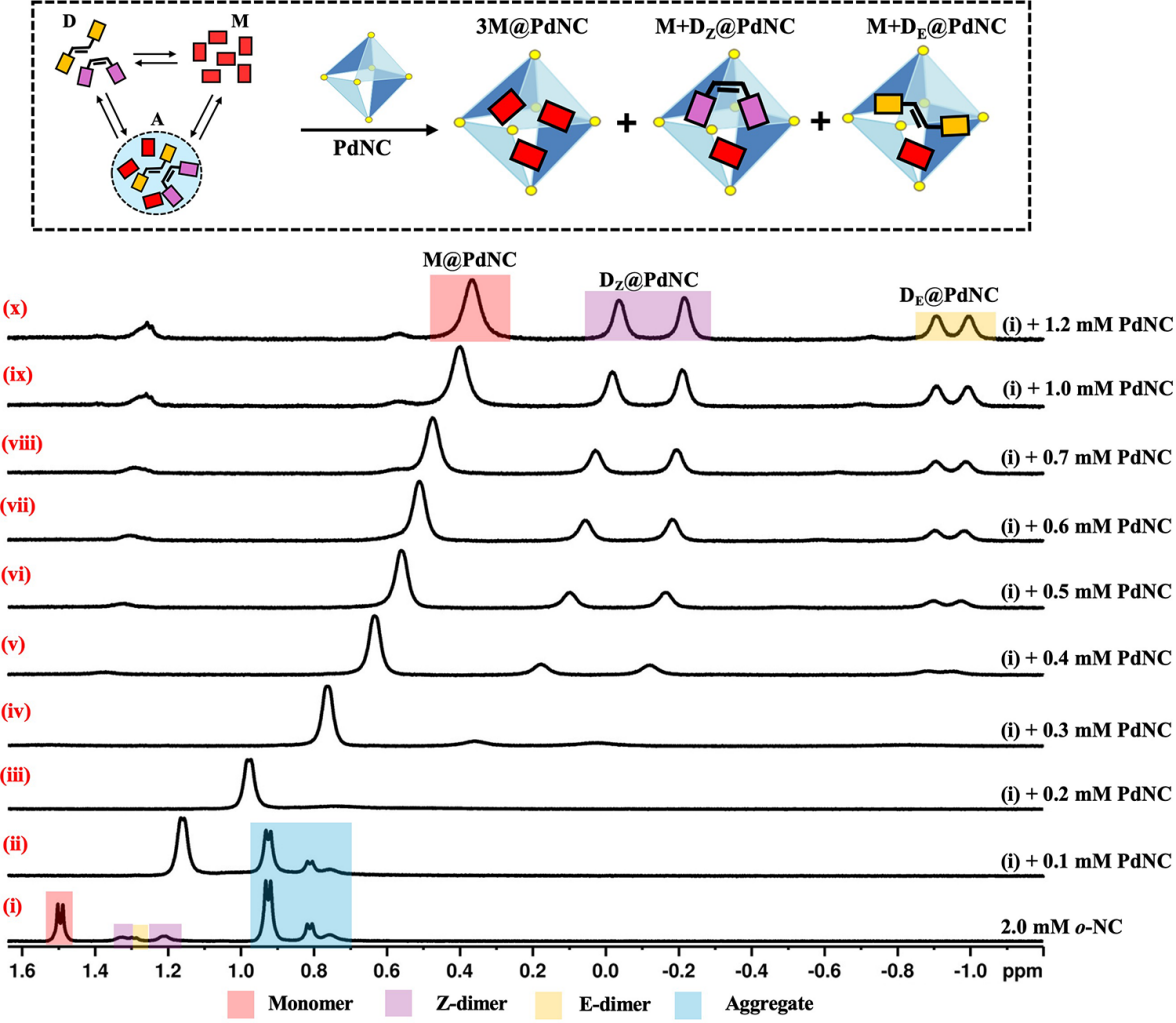
^1^H NMR titration spectra (500 MHz, D_2_O, 25 °C) of (i) 2.0 mM *o*-NC with (ii) 0.1, (iii) 0.2, (iv) 0.3, (v) 0.4, (vi) 0.5, (vii) 0.6, (viii) 0.7, (ix) 1.0, and (x) 1.2 mM PdNC. Addition of PdNC resulted in the disassembly of *o*-NC aggregates and encapsulation of both monomeric and dimeric species. Even with excess host present, the composition of the complexed guest remained unchanged.

**Figure 3:**
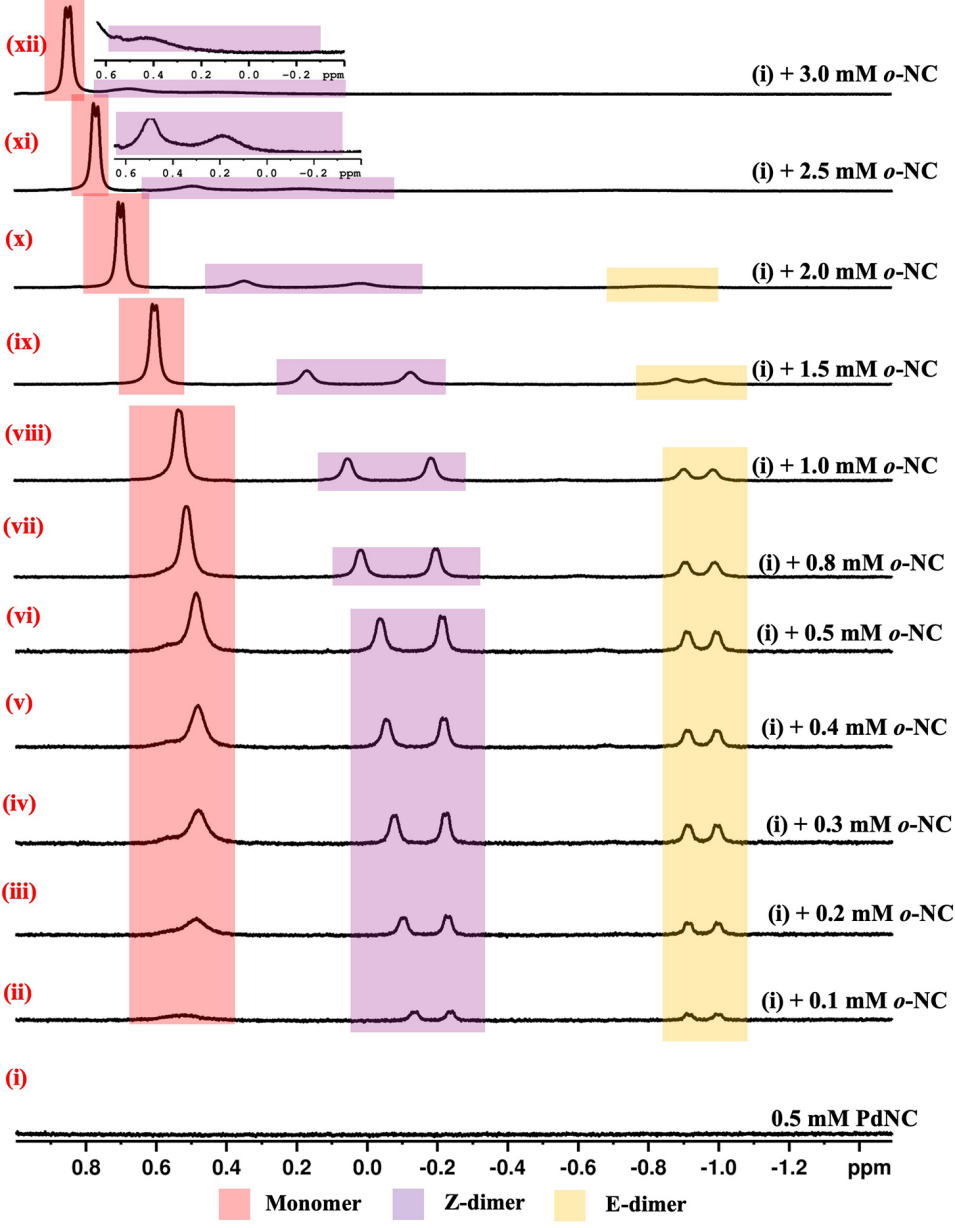
^1^H NMR titration spectra (500 MHz, D_2_O, 25 °C) showing (i) 0.5 mM PdNC host with increasing concentrations (ii–xii) 0.1–3.0 mM of the *o*-NC guest. Initial complex formation is evident from the presence of three guest species, whose signals broaden and shift to higher frequencies (deshielding) upon excess G:H > 3:1 due to dynamic exchange.

### Complexation of *o*-NC with PdNC—Addition of Guest to Host

The dynamic nature of the *o*-NC@PdNC complex is again evident when the titration is conducted by adding guest to host (Figure 3); the guest D_*Z*_ and D_*E*_ signals (assignments below) broaden at points beyond 3G:1H (>1.5 mM *o*-NC to 0.5 mM PdNC) and shift toward the free guest in water (Figure 3ix–xii). Clearly, when more than three guest molecules per cage are introduced into the solution, the bound guests exchange with free guests in water, resulting in a single signal rather than two independent signals for free and complexed guests, indicating fast exchange of these species in the NMR experiment. The three-guest capacity of PdNC binding is also indicated from the PdNC host H_a_ signal evolution, as shown in Figure S6. A broadening and shifting of H_a_ are observed as guest concentration is increased up to the three-guest capacity. We note that the dynamic H-G exchange observed for 3*o*-NC@PdNC is different from what was noted with CB8 and OA as hosts, which formed more stable complexes.^31^ An important point to note is that, even though the PdNC complex is dynamic, the guest *o*-NC remains mostly within the host.

The ^1^H NMR spectra recorded upon gradual addition of PdNC to a 2.0 mM solution of *o*-NC, as shown in Figure 2 (and Figures S4 and S5) display several notable features. Spectra corresponding to early additions of the host reveal that solution aggregates are dispersed and ultimately included as M and D within the PdNC cavity. A point to note is that it is known that salts, such as NaCl, will favor aggregation. In this case, the salt [PdNC]^12+^12­[NO_3_]^−^ shows the opposite behavior, namely, favoring deaggregation. This is likely due to the presence of the large cavity in the salt that can include the M and D structures. With increasing addition of PdNC, the signal due to M *i-*Pr methyls progressively shifts to a lower δ-value, suggesting a greater fraction of M complexed within the cage. Upon completion of the titration, only shielded signals due to M (δ 0.5 ppm) and one or more species with four signals between δ 0.1 and −1.0 ppm remain. No signals due to uncomplexed M and D in solution were seen.

### Assignment of NMR Signals of PdNC Complexed M, D_*Z*_, and D_*E*_

The four signals between δ 0.1 and −1.0 ppm are assigned as two nonequivalent (diastereotopic) methyl groups of D_*Z*_ and D_*E*_, as labeled in Figure 2x (vide infra). As discussed earlier, the nonplanarity and restricted rotation of the C–N bond in the dimers make the two isopropyl methyl groups nonequivalent. The first two signals near δ 0.1 and −0.3 ppm are assigned to D_*Z*_ and the two weaker signals at δ −0.8 and −1.1 ppm to D_*E*_. These assignments are supported by the greater temperature dependence observed in the D_*Z*_ population change, as shown in Figure 4 (and Figures S7 and S8 and Table S1). This observation is consistent with previous variable-temperature NMR studies across various solvents, including water, which document that D*_Z_* concentrations always increase more than D_*E*_ concentrations at lower temperatures.^23^ To confirm that these four ^1^H NMR signals are due to the dimers, the ^13^C NMR spectra and ^1^H–^13^C HSQC spectra were recorded (Figures S9–S12 and Table S2). As observed for the ^1^H NMR signals, the ^13^C signals of *i*-Pr methyl and methine carbons are shielded when encapsulated within PdNC (Figure S10 and Table S2). ^1^H–^13^C HSQC spectra shown in Figure S11, along with earlier assignments of ^13^C signals of *o*-NC M, D_*Z*_, and D_*E*_ in CDCl_3_ (Table S2),^23^ allow us to assign the carbon signals for the PdNC-included *o*-NC dimers. The proportional displacement of these ^13^C NMR signals with respect to those free in CDCl_3_ supports the assignment of the five methyl ^1^H NMR signals of Figure 2x to M and D_*Z*_ and D_*E*_ as indicated above (Figure S10). We believe that upon completion of the titration shown in Figure 2 (trace viii), most PdNC hosts contain three *o*-NC guests, present as M or D forms. This creates a scenario in which, on average, some cages contain 3M, some M+D_*Z*_, and some M+D_*E*_. Given the nature of the *o*-NC-PdNC complex, we expect a dynamic equilibrium between M and D within the cage. In addition to the exchange between the guests present within the cage and with free ones in water, there is intracage exchange between the D_*Z*_ and D_*E*_ (see below).

**Figure 4:**
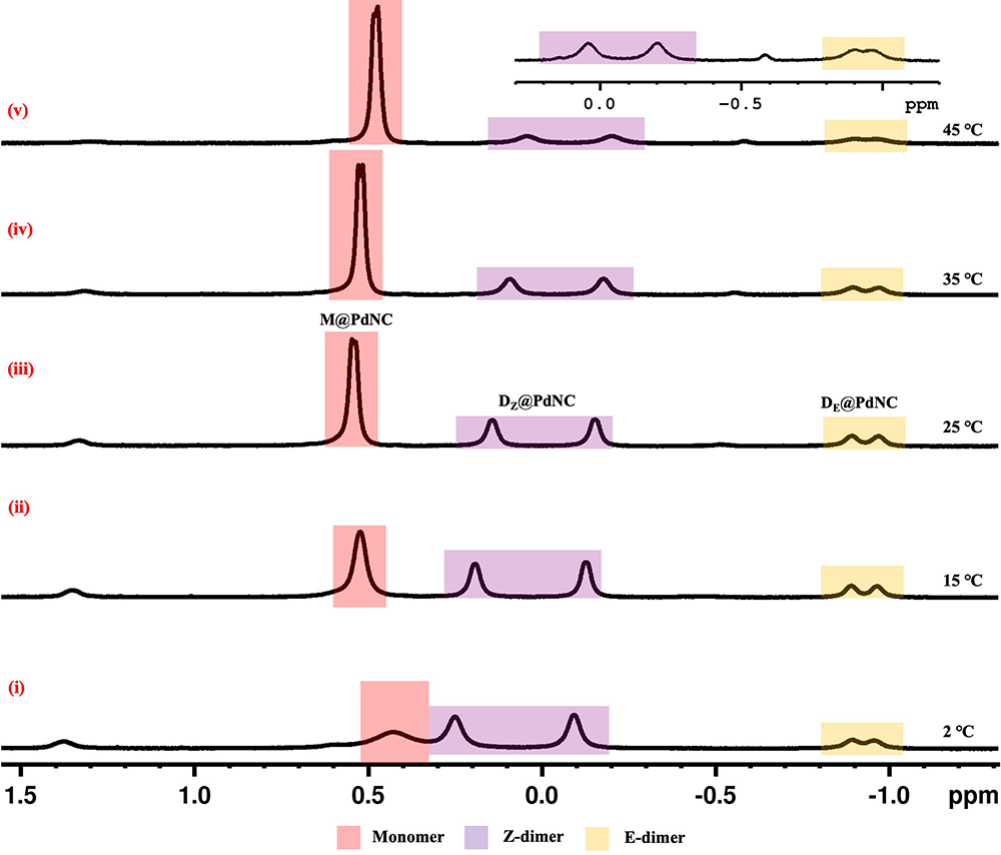
Variable-temperature ^1^H NMR (500 MHz, D_2_O) spectra of 0.7 mM PdNC complexed with 2.0 mM *o*-NC (isopropyl methyl protons) at (i) 2, (ii) 15, (iii) 25, (iv) 35, and (v) 45 °C. The inset in panel (v) highlights specific regions with greater intensity.

### Effect of PdNC Confinement on C–N (Phenyl) Rotation in *Z* and *E* Dimers

An important point to note is that the PdNC cage environment renders C–N (cumyl) rotation slow on the NMR time scale for both D_*Z*_ and D_*E*_ structures, giving rise to diastereotopic Me signals for the *i*-Pr groups in both dimers in the cage environment at ambient temperature, compared to only D_*Z*_ showing this behavior in water. An interesting feature relates to the influence of confinement on the rotational mobility of the C–N bond of *o*-NC. The spectra displayed in Figure 2 highlight interesting features related to the structures of the dimers in water and within PdNC. While the *i*-Pr methyl signals of D_*E*_ within PdNC appear as two singlets (*i*-Pr methyls are diastereotopic), in water, they appear as a single resonance (Figure S13). The resolution of diastereotopic methyl signals for D_*E*_ in PdNC is likely the result of confinement on the C–N rotations of *o*-NC, which interchanges these methyl environments. In the case of the D_*Z*_ isomer within PdNC, the *i*-propyl methyl NMR signal separation depends on the H:G ratio during the titration. For *o*-NC in water, this separation is independent of the concentration (Figure S13 and Table S3). Within PdNC, the D_*Z*_ methyl signal separation increases during the titration, and with excess *o*-NC beyond the cage capacity, these signals broaden and shift to higher ppm, which is an indication of dynamic exchange between caged and free guest under these conditions.

### Equilibrium between Free and PdNC Complexed M and D

Figures 2 and 3 reveal the dynamic features of PdNC binding of *o*-NC, which is especially evident during early stages of addition of host to the guest (Figure 2ii–vi) and at the later parts of titration of guest into the host solution (Figure 3ix–xii). These are conditions with >3 guest per host, giving a mixture of both bound and unbound guests, which have dramatically different NMR chemical shifts. As seen in Figure 2, the signal due to M continuously shifts at early stages of addition of PdNC to the *o*-NC solution. Following the initial disappearance of aggregates upon PdNC addition, the *i*-propyl methyl signals of D go through continuous shape and NMR shift changes until they emerge as four singlets (Figure 2vi). This signal evolution confirms that D goes in and out of the cavity at the rate dependent on the host:guest concentrations and ratios in water. Had there been no dynamic exchange during the NMR lifetime (ms), the D_*Z*_ and D_*E*_ as well as M signals would have emerged as sharp resonances at their final chemical shift values (Figure 2viii) from the beginning of the titration. Consequences of D exchange are more vivid during the addition of *o*-NC to the PdNC (Figure 3). When the *o*-NC concentration is increased beyond 3 equiv (more than 1.5 mM for 0.5 mM PdNC), the *i*-Pr methyl signals due to D_*E*_ become a broad singlet and the methyl signals for D_*Z*_ shift further apart, broaden, and become distorted (Figure 3ix–xii). This distortion and separation increase with an increased concentration of *o*-NC. In addition, the difference in ^1^H NMR shifts between the nonequivalent methyl signals of D_*Z*_ varies from 0.1 to 0.31 ppm (Table S5), while that for D_*E*_ varies only between 0.09 and 0.08 ppm before broadening into an unresolved singlet. We attribute this to the exchange of both dimers between the aqueous exterior and PdNC interior.

### Equilibrium between Complexed M and D within PdNC Cavity

Having identified that the PdNC can accommodate both M and D structures, we were curious about how this host would influence the M:D ratio. Direct comparison of the M:D ratio in water with and without PdNC at [*o*-NC] of 0.1–0.4 mM, where the solution contains no aggregates, reveals that the amount of D is much higher in the presence of PdNC than in its absence (Figure S13 and Table S4). This is a clear demonstration of how the microenvironment and local concentration within the PdNC host alters the 2 M ⇄ D equilibrium. It is also interesting to note that in contrast to PdNC, OA, which includes two molecules of *o*-NC, did not allow it to dimerize.^31^ The difference in behavior within OA and PdNC brings out the role of free space on reaction dynamics in supramolecular chemistry.^39^ One could view the chemistry occurring within the PdNC cage, as suggested by Cram et al. in the case of his hemicarcerand, to be the result of "a new phase of matter" coexisting with bulk water.^44^

### Temperature Dependence of Equilibrium between M and D within PdNC

In all three types of occupied PdNC complexes (3M, M+D_*Z*_, and M+D_*E*_), assuming there is enough free space, one would expect the temperature to influence the relative proportions of M and D, mirroring what is observed in isotropic solution. Higher temperature would be expected to favor M and lower temperature to favor D (with D_*Z*_ populations more favored than D_*E*_ populations). Temperature-dependent (2–45 °C) NMR spectra shown in Figure 4 (and Figures S7 and S8 and Table S1) support this expectation. Based on ^1^H NMR signal integration, using the PdNC signals as an internal standard, the distribution of *o*-NC forms occupying PdNC at 25 °C in D_2_O, to a first approximation, is 35% 3M, 45% M+D_*Z*_, and 20% M+D_*E*_. This estimation assumes a three *o*-NC occupancy for all PdNC hosts and further that all D_*Z*_ bound structures are M+D_*Z*_@PdNC and all D_*E*_ bound structures are M+D_*E*_@PdNC, with any remaining excess M being part of a 3M@PdNC assembly. As the temperature is lowered, dimerization is favored and at 2 °C PdNC is found as 82% M+D_*Z*_@PdNC and 18% M+D_*E*_@PdNC, with apparently no 3M cages present. Conversely, at higher temperature, e.g., at 45 °C, more M structures form, giving rise to a higher population of M-bound PdNC hosts and lower concentrations of cages with D_*Z*_ and D_*E*_ guests.

### Equilibrium between Free and PdNC Complexed M Revealed by the Second Host CB8

As mentioned above, the H/G titration NMR results summarized in Figures 2 and 3 imply that *o*-NC can dynamically exchange in/out of the PdNC cavity. To further evaluate this prospective guest exchange, we employed a second host, namely, CB8, to selectively complex free M in water (Figure 5).^31^ Upon addition of 0.2 mM of CB8 to a solution of 1.0 mM *o*-NC and 0.3 mM PdNC, a new *i*-Pr methyl NMR signal appears at δ 0.8 ppm, while at the same time, the intensity of the corresponding signals from PdNC-bound *o*-NC decreases (Figure 5 and Figure S14). Based on the known NMR spectrum of M@CB8, we assign the new signal to the *o*-NC monomer bound in CB8. Broadening of the M@PdNC signal as well as the new signal (M@CB8) suggests an exchange between M in these two hosts. During the titration, the NMR resonance of methyl for M@CB8 remains nearly constant although the resonance line narrows with increasing concentration of CB8. This indicates that most of the time M is trapped within either PdNC or CB8 cavity with free M being only slightly populated and being in fast exchange with its bound forms. This observation is also consistent with the diffusion coefficient^45,46^ of PdNC complexed M (M@PdNC), which is only slightly higher than that for the complexed dimers D@PdNC (Figures S15 and S16). While *k*_diff_ for M@PdNC is 1.86 ± 0.02 × 10^–10^ m^2^/s, those for D_*Z*_@PdNC and D_*E*_@PdNC are 1.53 ± 0.04 × 10^–10^ and 1.53 ± 0.11 × 10^–10^ m^2^/s, respectively. Full titration spectra shown in Figure S14 support the conclusion that as the concentration of M within the PdNC host depletes, due to its exit and capture by CB8, D present in PdNC cavity dissociates to 2 M and eventually all guests are sequestered by CB8 as M. Upon completion of the titration, only the methyl signal (δ 0.8 ppm) corresponding to M@CB8 remains. The fact that CB8 captures all M from PdNC suggests that it forms a stronger complex with *o*-NC than does the latter. This host-to-host exchange of *o-*NC also affects the guest’s structure form, converting M,D mixtures to pure M distributions, highlighting the significant and different host effects on the degree of dynamic covalent dimerization of *o*-NC in these environments.

**Figure 5:**
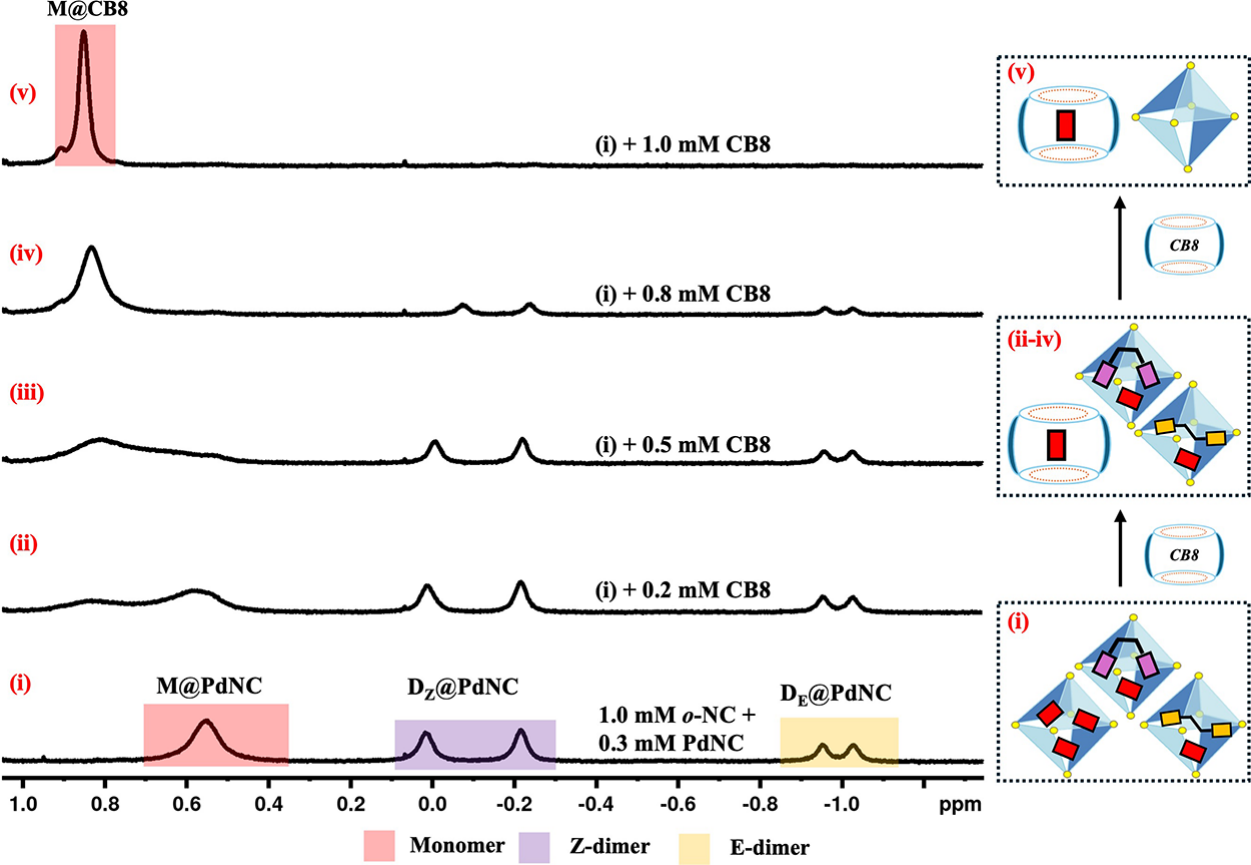
^1^H NMR partial titration spectra (800 MHz, D_2_O, 25 °C) of (i) 1.0 mM *o*-NC with 0.3 mM PdNC with added (ii) 0.2, (iii) 0.5, (iv) 0.8, and (v) 1.0 mM CB8.

Results thus far have established (a) PdNC accommodates up to three *o*-NC units in the form of M and D, (b) M and D captured within PdNC establish an equilibrium with free M in water as indicated by the capture of M by CB8, and (c) as indicated by temperature-dependent ^1^H NMR spectra PdNC, including M and D establish dynamic equilibrium between themselves within the cage. Apparently, in the absence of CB8, the equilibrium between complexed and free M is not detectable.

### Dynamics of M ⇄ D Equilibrium within PdNC as Probed by EXSY NMR

To gain an insight into the mechanism and timelines of the structural interconversions between M and D isomers in both caged and free environments, exchange spectroscopy (two-dimensional EXSY NMR)^19,47,48^ experiments of an aqueous solution of *o*-NC (2.0 mM) in the presence of PdNC (0.7 mM) at various mixing times (5–700 ms) and temperatures (5 and 25 °C) were performed. For comparison, similar experiments of *o*-NC (2.2 mM) at 25 °C in D_2_O solution without PdNC were also conducted. The 2D EXSY data presented in Figure 6 and Figures S17–S25 can be summarized as follows. (a) In D_2_O solution (2.2 mM *o*-NC) without PdNC at 25 °C, no solution phase 2 M ⇄ D exchange is observed at ≤500 ms mixing time (Figures S17–S19). However, at longer mixing time (700 ms) and higher concentration (2.4 mM), M ⇄ D exchange is observed.^37^ No direct exchange between D_*Z*_ and D_*E*_ is observed in D_2_O or in CDCl_3_.^23^ (b) Within the PdNC environment, two types of NMR signal interchanges for *o-*NC are observed: those between M and D isomers and, surprisingly, also those between the two isomeric dimers (D_*Z*_ and D_*E*_). (c) At 25 °C and 20 ms mixing time, there are exchange signals between M and D (both isomers) as well as between D_*Z*_ and D_*E*_ (Figure 6a). (d) Shortening the mixing time to 5 ms at 25 °C or lowering the temperature to 5 °C (Figure 6b,c) results in a detectable exchange only between D*_Z_* and D_*E*_. This indicates the D-to-D transformation to be the fastest exchange process in the PdNC. (e) At 5 °C and 5 ms mixing time, the system appears frozen, exhibiting no observable exchange signals between any species (Figure 6d).

**Figure 6:**
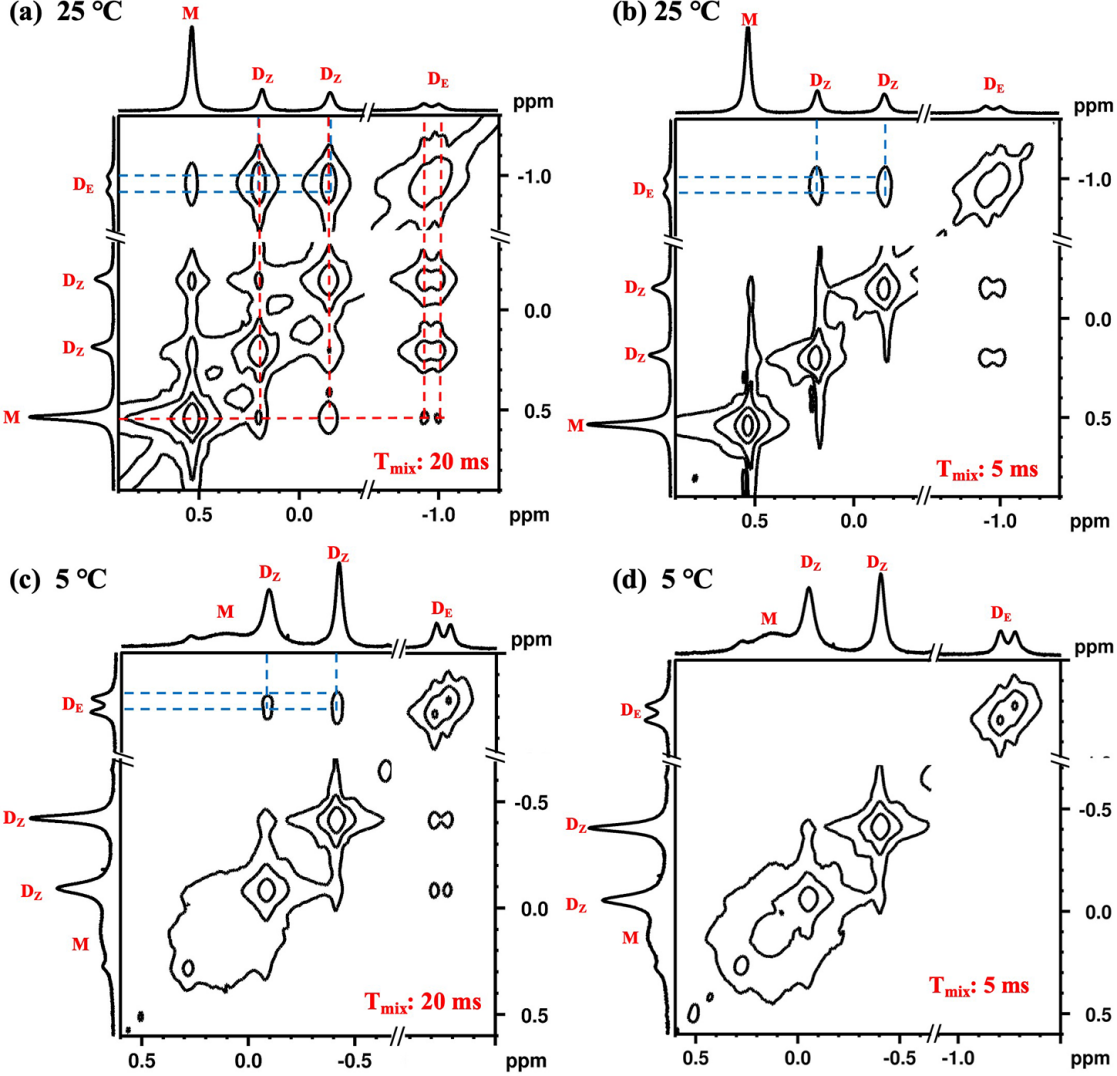
2D ^1^H–^1^H EXSY (800 MHz, D_2_O) spectra showing exchange signals between isopropyl methyl groups in a solution containing 2.0 mM *o*-NC and 0.7 mM PdNC at 25 °C (a, b) and 5 °C (c, d). Red dashed lines in panel (a) indicate exchange signals between complexed M and Ds, while blue dashed lines in panels (a)–(c) indicate exchange signals between D_*Z*_ and D_*E*_. Mixing time (*T*_mix_) is indicated in each panel.

### Mechanism of D_*Z*_ ⇄ D_*E*_ Exchange in Solution

The most interesting observation is the direct interconversion between the isomers of the dimer within PdNC. Prior studies (using EXSY NMR) have shown that the D*_Z_* ⇄ D_*E*_ exchange proceeds only via a dissociation (D-to-2M) and association (2M-to-D) pathway in solution for nitrosobenzene^19^ and for *o*-NC in CDCl_3_ ^1^ and in D_2_O.^23^ The alternate mechanism involving geometric *N*,*N* rotational isomerization has been ruled out based on the absence of direct EXSY correlation between the two D isomers in isotropic solution. This mechanistic conclusion has also been supported on computational^23^ and theoretical grounds.^11–13^ In the case of *o*,*o′*-azodioxytoluene in acetonitrile, the rotational process was explicitly considered and ruled out on the basis of thermodynamic and activation parameters.^49^ Thus, in solution, rotation around the N=N bond of the azodioxide dimer is believed generally to require higher energy and be slower than D dissociation to 2M, thus negating the direct interconversion mechanism of D_*Z*_ and D_*E*_ exchange.^4,50^

### Likely Mechanism of D_*Z*_ ⇄ D_*E*_ Exchange within PdNC

Recognizing that the mechanism could change when the reactant molecules are sequestered in a small space,^51,52^ we consider both pathways for D_*Z*_ and D_*E*_ interconversion within the "confined" environment of the PdNC. In the cage, the reactant molecules are segregated in a restricted environment, with the available free space around them limited. Under these conditions, in addition to thermodynamic factors, the availability of "free space" may play a role in determining the preferred mechanistic pathway.^39^ Scheme 2 illustrates the possible D_*Z*_ ⇄ D_*E*_ exchange pathways.

**Scheme 2:**
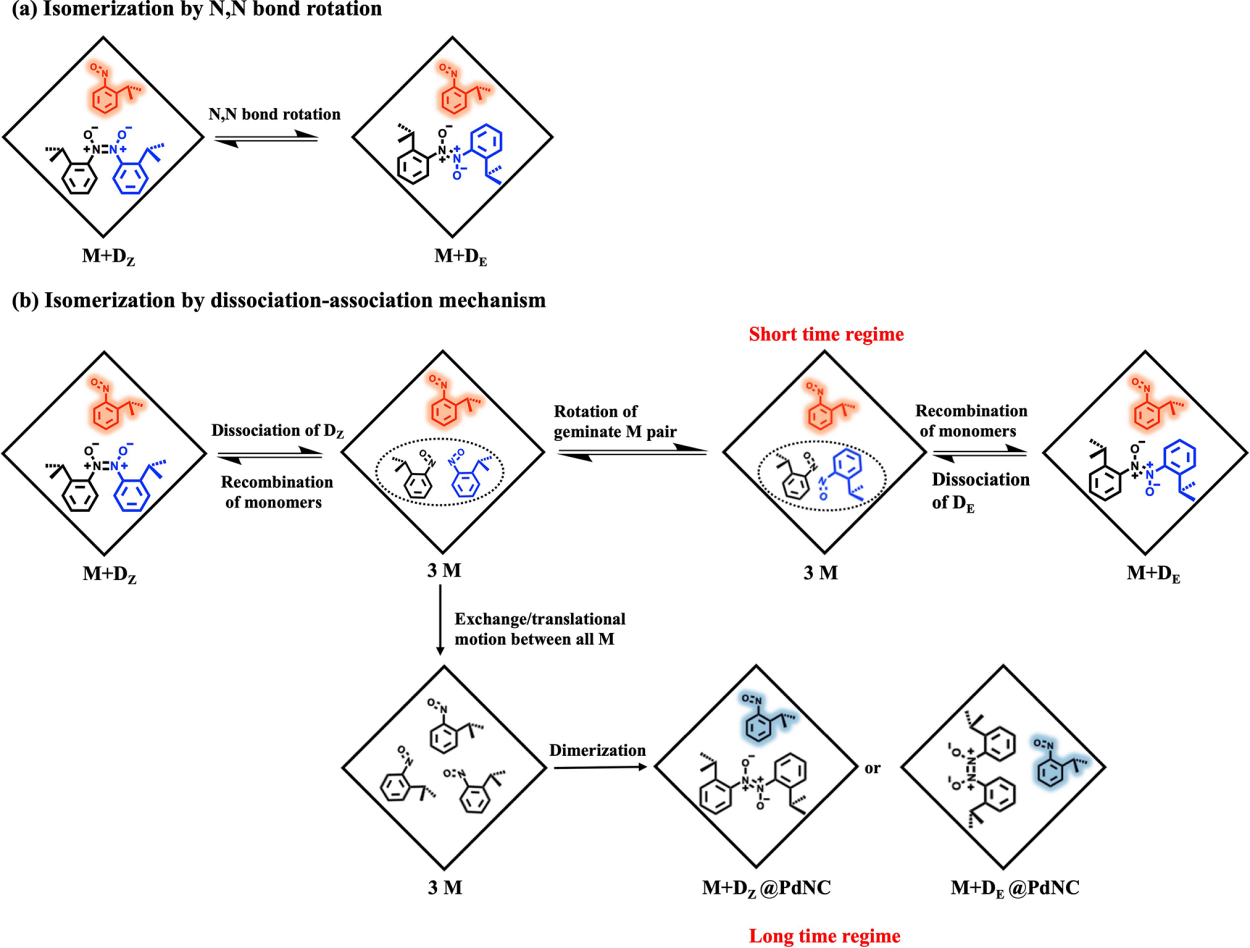
Schematic Representation of Two Isomerization Pathways: (a) via *N*,*N*-Bond Rotation and (b) via Dissociation–Association Mechanisms^a^

a For the dissociation–association pathways, exchange processes at the short-time regime (mixing time in EXSY NMR *T*_mix_ ≤ 10 ms) and the long-time regime (*T*_mix_ ≥ 10 ms) at 25 °C are depicted. Shading around *o*-NC structures indicates the exchanging monomer, and the dotted circle represents the geminate M pair formed from D. The square denotes the cavity of the PdNC nanoreactor, within which distinct exchange processes are observed.

From the EXSY results alone, we cannot rule out an *N*,*N* rotational mechanism of D_*Z*_ ⇄ D_*E*_ interconversion within PdNC, which could become more favored in the restricted (entropy limiting) space (Scheme 2a). However, it is perhaps most likely (in keeping with known nitrosobenzene dimer isomerization pathways as mentioned above) that the dissociation/association pathway of dimer interconversion remains operational in the PdNC host and yields the observed EXSY results due to the confined space, as illustrated in Scheme 2b (vide infra).

In Scheme 2, the PdNC nanoreactor is represented as a square where the dynamic covalent interchanges occur. In Scheme 2b, the dotted circle within the cavity represents a "geminate" M pair, formed by dissociation of D, within restricted conformational space. The "free" M within the cavity is represented by red/blue-shaded structures. We propose that the difference in freedom experienced by "free" M in PdNC and the favorably oriented "geminate" M pairs in PdNC accounts for the observations summarized above.^53–55^ The occurrence of exchange between M and D as well as between D_*Z*_ and D_*E*_ at 25 °C with 20 ms mixing time can be accounted for if, in this time window, D dissociates to 2 M, and the resulting M pair separates far enough to equilibrate with M in the PdNC, which then recombine to regenerate D in both isomeric forms (Figure 6a and Scheme 2b, lower track). Thus, the M pair derived from D, although confined within the PdNC cavity, undergoes both translational and rotational motions to scramble all M’s in the cage in 20 ms mixing time. Shortening the time window to 5 ms mixing time at 25 °C results in the loss of exchange between M and D but not between D_*Z*_ and D_*E*_ (Figure 6b and Scheme 2b, upper track). This is most likely the result of loss of translational mobility but not rotational mobility in the shorter time window. We envision that in 5 ms, the geminate M pair formed from D dissociation move far enough to allow rotation of one of the M molecules but not far enough to become "free" and scramble all M in the cage. The geminate pair may recombine with or without rotation to reform D_*Z*_ or D_*E*_. This process supports EXSY correlation between the signals of the isomeric dimers but not between M and D in the PdNC host. Importantly, at 5 °C and 5 ms mixing time, there are no exchange signals detected (Figure 6d). Although we favor the presented geminate pair recombination pathway as the simplest way to accommodate all observed data at this time, we recognize that we cannot rule out from existing data the direct rotational pathway.

## Conclusions

In this study, we have demonstrated that the *o*-NC complex dynamic mixture of M, D, and A structures is altered not only by temperature and solvent polarity but also by inclusion within a water-soluble host. Interestingly, both M and D included within PdNC establish an equilibrium among themselves within the restricted space, which also enhances their rate of interchange. The restricted molecular motions (translational and rotational) in the PdNC confined space introduce a new dynamic pathway for the molecular 2 M ⇄ D transformations. Strikingly, at shorter time scales, direct D-to-D exchange interconversion between D_*Z*_ and D_*E*_ isomers, which does not happen in solution, is detected by ^1^H–^1^H EXSY NMR experiments in the PdNC nanoreactor. The restricted space of the PdNC is also sufficient to (a) distinguish diastereotopic Me NMR signals of the D_*E*_ isomer not distinguished in solution and (b) results in the formation of significant amounts of D_*Z*_ and D_*E*_ structures at low *o*-NC concentrations in water, which give only trace dimer in the absence of this host.

It is interesting to compare the structure and dynamics of the complexes of *o*-NC with β-CD, CB8, OA,^9^ and the PdNC reported here. From the aqueous solution containing a mixture of M, D, and A, hosts β-CD, CB8, and OA selectively include only M. While the complexes of β-CD and CB8 are dynamic, similar to the PdNC complex, the OA host forms a strong static 2:2 capsuleplex. While both OA and PdNC include more than one molecule of M, the former does not allow dimerization. The larger cage dimension and consequently more free space of the PdNC allow more freedom for the included guest M and favor dimerization. However, the presence of four openings renders complex dynamics similar to CD and CB complexes, which have two openings. This study, combined with our earlier reports on cyclodextrins, cucurbituril, and octa acid hosts,^9^ demonstrates the value of utilizing several different kinds of host cavities to achieve and exploit subtle supramolecular features of host–guest assembly, including effects on guest reaction dynamics. Watching molecules dance within a confined space of a nanoreactor is intellectually stimulating, challenging, and fun. The knowledge we gain from such a study can provide insight into biological events where small molecules dance on a stage created by enzyme pockets or biological machines.

## Experimental Section

### Materials and Methods

NMR experiments, including titration, characterization, DOSY, EXSY, and NOESY measurements, were performed on Bruker Avance NMR spectrometers operating at 800 and 500 MHz (both equipped with cryoprobes). NMR shifts are reported in parts per million (ppm) relative to the residual HDO peak at δ = 4.79 ppm for ^1^H NMR. Structural assignments were determined using additional information from gs-COSY, NOESY, EXSY, DOSY, and gs-HSQC experiments. Guests (*o*-nitrosocumene and *o*-nitrocumene) were synthesized according to reported procedures.^23,56^ The host PdNC was sourced from Wako Chemicals USA Inc. and used as received.

### Sample Preparation for Host–Guest Inclusion Studies Using NMR Spectroscopy

NMR titration experiments were performed to investigate host–guest complexation using a Palladium nanocage (PdNC) host (Scheme 1).^33,34^ The general procedure involved the sequential addition of guest to a solution of the PdNC prepared in 0.6 mL of D_2_O, or vice versa, followed by the acquisition of ^1^H NMR spectra after each addition to monitor changes in both host and guest NMR signals.

#### Procedure A (Guest → Host)

A PdNC solution was prepared at 0.5 mM in D_2_O. A stock solution of the guest (*o*-NC) was prepared at 60.0 mM in DMSO-*d*_6_. For titration, variable equivalents of *o*-NC were added sequentially to the PdNC solution. To account for dilution with guest addition, the volume of guest added at each step was included in the total volume of D_2_O when calculating concentrations. After each addition, the NMR tube was vigorously shaken to ensure complete mixing, and a ^1^H NMR spectrum was recorded.

#### Procedure B (Host → Guest)

A guest solution of *o*-NC was prepared at 2.0 mM in 0.6 mL of D_2_O. Variable equivalents of PdNC were added sequentially from a stock solution prepared at 10.0 mM in D_2_O. To account for dilution with PdNC addition, the volume of PdNC added at each step was included in the total volume of D_2_O when calculating concentrations. After each addition, the NMR tube was vigorously shaken to ensure complete mixing, and a ^1^H NMR spectrum was recorded.

## Supplementary Material



## Data Availability

The data underlying this study are available in the published article and its Supporting Information.

## Notes

^1^H NMR titration spectra of *o*-NC and *o*-nitrocumene with PdNC, VT NMR, and ^13^C NMR characterization of the host–guest complex; 2D NMR (COSY, NOESY, HSQC, and DOSY) and EXSY spectra of the *o*-NC and *o*-NC@PdNC complex at various mixing times (PDF)

## References

[1] Gowenlock B. G., Richter-Addo G. B. (2008). The First 85 Years of C-Nitroso Compounds: A Survey
of the Salient Features. J. Chem. Educ..

[2] Gowenlock B. G., Richter-Addo G. B. (2005). Dinitroso
and Polynitroso Compounds. Chem. Soc. Rev..

[3] Vančik, H. , Aromatic C-Nitroso Compounds; Springer: Heidelberg, 2013.

[4] Beaudoin D., Wuest J. D. (2016). Dimerization of Aromatic C-Nitroso
Compounds. Chem. Rev..

[5] Lin F., Tang R., Liu S., Tan Y. (2025). Recent Advances in
the Synthetic Applications of Nitrosoarene Chemistry. Org. Biomol. Chem..

[6] Gao Y., Yang S., Xiao W., Nie J., Hu X.-Q. (2020). Radical
Chemistry of Nitrosoarenes: Concepts, Synthetic Applications and Directions. Chem. Commun..

[7] Molander G. A., Cavalcanti L. N. (2012). Nitrosation of Aryl and Heteroaryltrifluoroborates
with Nitrosonium Tetrafluoroborate. J. Org.
Chem..

[8] Beaudoin D., Maris T., Wuest J. D. (2013). Constructing
Monocrystalline Covalent
Organic Networks by Polymerization. Nat. Chem..

[9] Biljan I., Vančik H. (2025). Aromatic C-Nitroso
Compounds: From Solid-State Reactivity
to New Materials. ChemistrySelect.

[10] Bianchi P., Monbaliu J.-C. M. (2021). Three Decades of Unveiling the Complex Chemistry of
C-Nitroso Species with Computational Chemistry. Org. Chem. Front..

[11] Varga K., Biljan I., Tomišić V., Mihalić Z., Vančik H. (2018). Quantum Chemical Calculations of Monomer–Dimer
Equilibria of Aromatic C-Nitroso Compounds. J. Phys. Chem. A.

[12] Rončević I., Bibulić P., Vančik H., Biljan I. (2018). Solution Equilibria
of Aromatic Dinitroso Compounds: A Combined NMR and DFT Study. Struct. Chem..

[13] Minato T., Yamabe S., Oda H. (1982). A Theoretical Study
on the *cis/trans* Isomerization of Azodioxymethane. Can. J. Chem..

[14] Glaser R., Murmann R. K., Barnes C. L. (1996). Why Do Nitroso Compounds Dimerize
While Their Oxime Tautomers Do Not? A Structural Study of the *Trans*-Dimer of 2-Chloro-2-methyl-3-nitrosobutane and Higher
Level ab Initio Study of Thermodynamic Stabilities and Electronic
Structures of Isomers of Diazene Dioxides. J.
Org. Chem..

[15] Fehling C., Friedrichs G. (2011). Dimerization
of HNO in Aqueous Solution: An Interplay
of Solvation Effects, Fast Acid-Base Equilibria, and Intramolecular
Hydrogen Bonding?. J. Am. Chem. Soc..

[16] Bunce N. J. (1978). Fluorescence
from an Upper Singlet of Aromatic C-Nitroso Compounds. Chem. Phys. Lett..

[17] Condirston D. A., Knight A. R., Steer R. P. (1980). Do Aromatic C-Nitroso
Compound Fluoresce
from Their Second Excited Singlet States?. J.
Photochem..

[18] Chernoff D. A., Hochstrasser R. M. (1980). Comment on “Fluorescence from an Upper Singlet
of Aromatic C-Nitroso Compounds”. Chem.
Phys. Lett..

[19] Orrell K. G., Šik V., Stephenson D. (1987). Study of the Monomer-Dimer Equilibrium
of Nitrosobenzene Using Multinuclear One-and Two-Dimensional NMR Techniques. Magn. Reson. Chem..

[20] Orrell K. G., Stephenson D., Rault T. (1989). NMR Study of the Monomer—Dimer
Equilibria of Dimethylnitrosobenzenes in Solution. Identification
of Mixed Azodioxy Dimeric Species. Magn. Reson.
Chem..

[21] Orrell K. G., Stephenson D., Verlaque J. H. (1990). Monomer–Dimer Solution Equilibria
of 2, 4, 6-Trialkylnitrosobenzenes and 2, 4, 6-Trialkylnitrosobenzene/Nitrosobenzene
Mixtures. A Study Using One-and Two-Dimensional NMR Techniques. J. Chem. Soc. Perkin Trans. 2.

[22] Fletcher D. A., Gowenlock B. G., Orrell K. G. (1998). Structural Investigations of C-Nitrosobenzenes.
Part 2. NMR Studies of Monomer–Dimer Equilibria Including Restricted
Nitroso Group Rotation in Monomers. J. Chem.
Soc., Perkin Trans. 2.

[23] Rogers C. H., Pradeep A., Galiano L. A., Kelley S. A., Varadharajan R., Belmore K., Whitt L. M., Li Y., Champagne P. A., Ramamurthy V., Blackstock S. C. (2024). Dynamic
Covalent and Noncovalent
Assembly of *o*-Nitrosocumene in Organic Solvents and
Water. Chem. Commun..

[24] Pike S. J., Heliot A., Seaton C. C. (2020). *ortho*-Substituent
Effect on the Crystal Packing and Solid State Speciation of Aromatic
C-Nitroso Compounds. CrystEngComm.

[25] Varga K., Lešić N., Bogović B., Pisačić M., Panić B., Biljan I., Novak I., Vančik H. (2019). Thermally-Induced
Reactions of Aromatic Crystalline Nitroso Compounds. ChemistrySelect.

[26] Rodenbough P. P., Karothu D. P., Gjorgjieva T., Commins P., Hara H., Naumov P. (2018). Reversible Photolysis of Nitrosobenzene *cis*-Dimer Monitored in Situ by Single Crystal Photocrystallography. Cryst. Growth Des..

[27] Bibulić P., Rončević I., Varga K., Mihalić Z., Vančik H. (2016). Structure and Topochemistry of Azodioxide Oligomers
in Solid State. J. Mol. Struct..

[28] Varga K., Vančik H. (2016). Topochemical
Effect in Thermal E–Z Isomerization
of Azodioxides in Solid State. J. Phys. Org.
Chem..

[29] Halasz I., Mestrovic E., Cicak H., Mihalic Z., Vancik H. (2005). Solid-State
Reaction Mechanisms in Monomer–Dimer Interconversions of *p*-Bromonitrosobenzene. Single-Crystal-to-Single-Crystal
Photodissociation and Formation of New Non-van der Waals Close Contacts. J. Org. Chem..

[30] Vancik H., Šimunić-Mežnarić ´ V., Ćaleta I., Meštrović E., Milovać S. (2002). Solid State
Photochromism and Thermochromism in Nitroso Monomer-Dimer Equilibrium. J. Phys. Chem. B.

[31] Pradeep A., Rogers C. H., Varadharajan R., Kelley S. A., Marek R., Blackstock S. C., Ramamurthy V. (2025). Supramolecular Confinement as a Tool
to Control the Dynamic Molecular Assembly of *o*-Nitrosocumene
in Water. Chem. Commun..

[32] Varadharajan R., Kelley S. A., Jayasinghe-Arachchige V. M., Prabhakar R., Ramamurthy V., Blackstock S. C. (2022). Organic
Host Encapsulation Effects
on Nitrosobenzene Monomer–Dimer Distribution and C–NO
Bond Rotation in an Aqueous Solution. ACS Org.
Inorg. Au.

[33] Takezawa H., Fujita M. (2021). Molecular Confinement Effects by Self-Assembled Coordination
Cages. Bull. Chem. Soc. Jpn..

[34] Yoshizawa M., Klosterman J. K., Fujita M. (2009). Functional Molecular Flasks: New
Properties and Reactions within Discrete, Self-Assembled Hosts. Angew. Chem., Int. Ed..

[35] Brinker, U. H. ; Mieusset, J.-L. , Molecular Encapsulation. John Wiley & Sons, Ltd.: Chichester, UK, 2010.

[36] Ramamurthy, V. , Photochemistry in Organized and Constrained Media. VCH: New York, 1991.

[37] Balzani, V. ; Scandola, F. , Supramolecular Photochemistry; Ellis Horwood Ltd: Chichester, 1990; p 427.

[38] Cohen M. D. (1975). The Photochemistry
of Organic Solids. Angew. Chem. Int. Ed..

[39] Weiss R. G., Ramamurthy V., Hammond G. S. (1993). Photochemistry in Organized and Confining
Media: A Model. Acc. Chem. Res..

[40] Fletcher D. A., Gowenlock B. G., Orrell K. G. (1997). Structural Investigations of C-Nitrosobenzenes.
Part 1. Solution State ^1^H NMR Studies. J. Chem. Soc., Perkin Trans. 2.

[41] Kusukawa T., Fujita M. (1998). Encapsulation of Large,
Neutral Molecules in a Self-Assembled
Nanocage Incorporating Six Palladium (II) Ions. Angew. Chem., Int. Ed..

[42] Inokuma Y., Yoshioka S., Fujita M. (2010). A Molecular
Capsule Network: Guest
Encapsulation and Control of Diels–Alder Reactivity. Angew. Chem., Int. Ed..

[43] Yoshizawa M., Tamura M., Fujita M. (2006). Diels-Alder in Aqueous Molecular
Hosts: Unusual Regioselectivity and Efficient Catalysis. Science.

[44] Cram D. J., Tanner M. E., Thomas R. (1991). The Taming of Cyclobutadiene. Angew. Chem., Int. Ed. Engl..

[45] Cohen Y., Avram L., Frish L. (2005). Diffusion
NMR Spectroscopy in Supramolecular
and Combinatorial Chemistry: An Old Parameter—New Insights. Angew. Chem., Int. Ed..

[46] Morris K. F., Johnson C. S. (1992). Diffusion-Ordered Two-Dimensional
Nuclear Magnetic Resonance Spectroscopy. J.
Am. Chem. Soc..

[47] Jeener J., Meier B. H., Bachmann P., Ernst R. R. (1979). Investigation of
Exchange Processes by Two-Dimensional NMR Spectroscopy. J. Chem. Phys..

[48] Perrin C. L., Dwyer T. J. (1990). Application of Two-Dimensional NMR to Kinetics of Chemical
Exchange. Chem. Rev..

[49] Azoulay M., Wettermark G. (1978). Kinetics of
the Dissociation and *Cis-Trans* Isomerization of *o*, *o*’-Azodioxytoluene
(Dimeric *o*-Nitrosotoluene). Tetrahedron.

[50] Wajer T. A. J. W., de Boer T. J. (1972). C-Nitroso Compounds. Part XXIII: *Cis/Trans*-Isomerisation of Aliphatic Azodioxy Compounds (Dimeric Nitrosoalkanes). Recueil des Travaux Chimiques des Pays-Bas.

[51] Otolski C. J., Raj A. M., Ramamurthy V., Elles C. G. (2020). Spatial Confinement
Alters the Ultrafast Photoisomerization Dynamics of Azobenzenes. Chem. Sci..

[52] Ramamurthy V. (2015). Photochemistry
within a Water-Soluble Organic Capsule. Acc.
Chem. Res..

[53] Noyes R. M. (1955). Kinetics
of Competitive Processes When Reactive Fragments Are Produced in Pairs. J. Am. Chem. Soc..

[54] Rabinowitch E., Wood W. (1936). The Collison Mechanism and the Primary Photochemical Process in Solutions. Trans. Faraday Soc..

[55] Turro N. J., Cherry W. R. (1978). Photoreactions in Detergent Solutions. Enhancement
of Regioselectivity Resulting from the Reduced Dimensionality of Substrates
Sequestered in a Micelle. J. Am. Chem. Soc..

[56] Reddy K. R., Maheswari C. U., Venkateshwar M., Kantam M. L. (2009). Selective Oxidation
of Aromatic Amines to Nitro Derivatives using Potassium Iodide-*tert*-Butyl Hydroperoxide Catalytic System. Adv. Synth. Catal..

