# Supplementary material for: Controlling Chemical Dynamics of Molecular Assemblies through Nanoconfinement: o‑Nitrosocumene@Pd Nanocage
Source: J Org Chem. 2026 Jul 7;91(28):9679–90. doi: 10.1021/acs.joc.6c00239 (PMC13386535; doi:10.1021/acs.joc.6c00239)
Supplement: Supplementary file 1 [file jo6c00239_si_001.pdf]

Supporting Information

Controlling Chemical Dynamics of Molecular Assemblies Through Nanoconfinement: *o*-Nitrosocumene@Pd nanocage

Anu Pradeep,[a] Cory H. Rogers,[b] Radek Marek,[c,d,*] Silas C. Blackstock,[b,*]
Vaidhyanathan Ramamurthy [a,*]

a. Department of Chemistry, University of Miami, Coral Gables, Florida 33146

b. Department of Chemistry, The University of Alabama, Tuscaloosa, AL 35487-0336

c. National Center for Biomolecular Research, Faculty of Science, Masaryk University, Kamenice 5, Brno, 62500 Czech Republic

d. Department of Chemistry, Faculty of Science, Masaryk University, Kamenice 5, Brno, 62500 Czech Republic

	**Contents**	**Pg. No.**
	**NMR of *o*-NC**	
Fig S1	^{1}H NMR of *o*-NC in DMSO-d_6	S3
	**Studies of *o*-nitrocumene with PdNC**	
Fig S2-S3	^{1}H-NMR titration spectra of *o*-nitrocumene with PdNC (H → G)	S4-S5
	**Studies of *o*-NC with PdNC**	
Fig S4	^{1}H-NMR titration spectra of *o*-NC with PdNC (H → G)	S6
Fig S5	A plot of chemical shift of complexed guest vs PdNC concentration	S7
Fig S6	^{1}H-NMR titration spectra of *o*-NC with PdNC (G → H)	S8
Fig S7-S8	Variable temperature ^{1}H NMR spectra of *o*-NC@PdNC complex	S9, S11
Table S1	NMR integrals of complexed M & D from VT NMR	S10
Fig S9-S10	^{13}C NMR spectrum of *o*-NC@PdNC complex	S12-S13
Table S2	Chemical shifts of M &D in $CDCl_3$ vs PdNC	S13
Fig S11	^{1}H -^{13}C HSQC spectrum of PdNC complexed *o*-NC	S14
Fig S12	2D COSY spectrum of PdNC complexed *o*-NC	S15
Fig S13	^{1}H NMR spectra of *o*-NC at different concentrations	S16

Table S3	Chemical shift difference between *i*-Pr methyls of dimers	S17
Table S4	Distribution of *o*-NC M and D species in water vs PdNC	S17
Fig S14	^{1}H-NMR spectra of adding CB8 to *o*-NC@PdNC complex	S18
Fig S15-S16	2D DOSY spectrum of free PdNC, *o*-NC@PdNC	S19-S20
Fig S17-S19	2D NOESY of free *o*-NC at different mixing times at 25 ℃	S21-S23
Fig S20-S22	2D NOESY spectrum of *o*-NC@PdNC at 25 ℃	S24-S26
Fig S23	Plot of 2D integral value vs exchange buildup at different mixing	S27
Fig S24	2D NOESY spectrum of *o*-NC@PdNC at 5 ℃	S28
Fig S25	Plot of 2D integral value vs exchange buildup at different mixing time	S29
Table S5	Chemical shift differences of isopropyl methyls of both dimers in the presence of PdNC	S30
Fig S26	B3LYP-631G optimized structures of dimers D_Z and D_E & Crystal structure of D_Z	S31-S35

^{1}H NMR of *o*-NC in DMSO-d_6

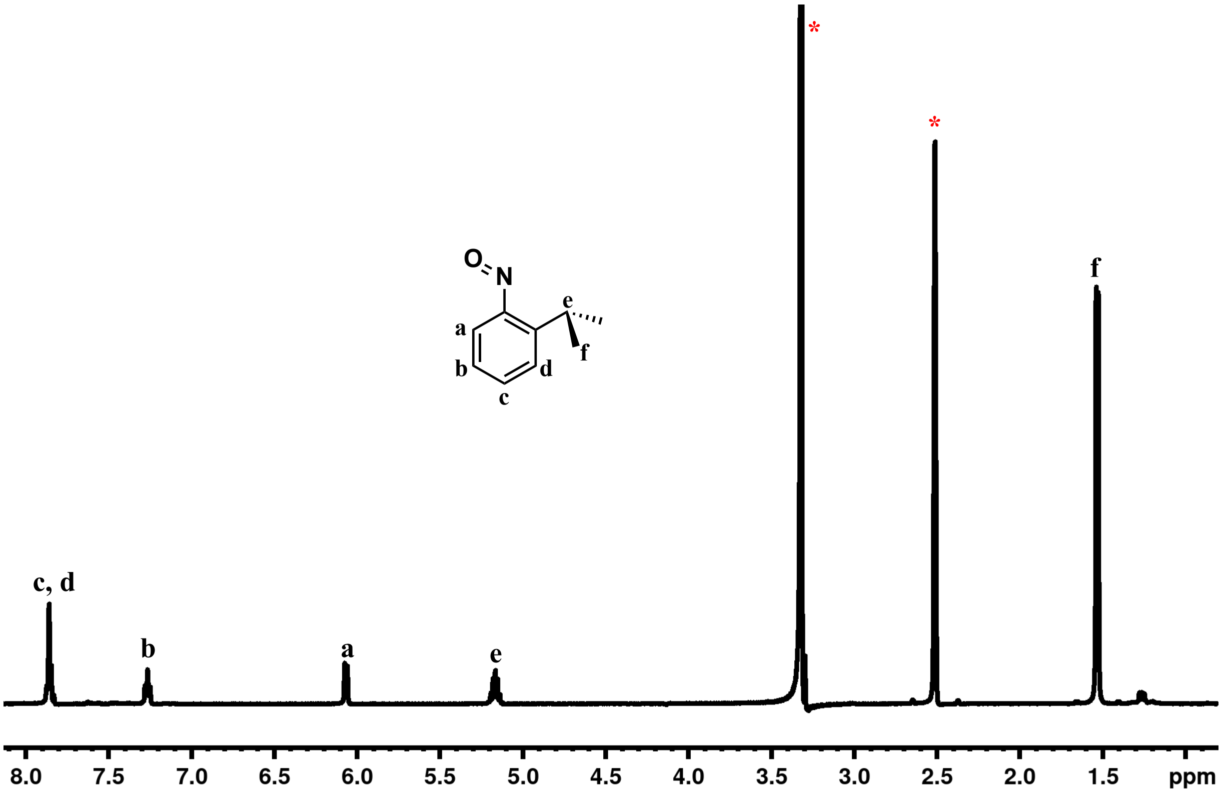

Figure S1:^{1}H NMR (500 MHz, DMSO-d_6, 25 °C) spectrum showing a 60 mM *o*-NC stock solution. The sample was prepared in a sealed capillary tube with 0.6 mL of DMSO-d_6. The * indicates residual solvent and water.

Encapsulation of *o*-nitrocumene with PdNC (Aliphatic region)

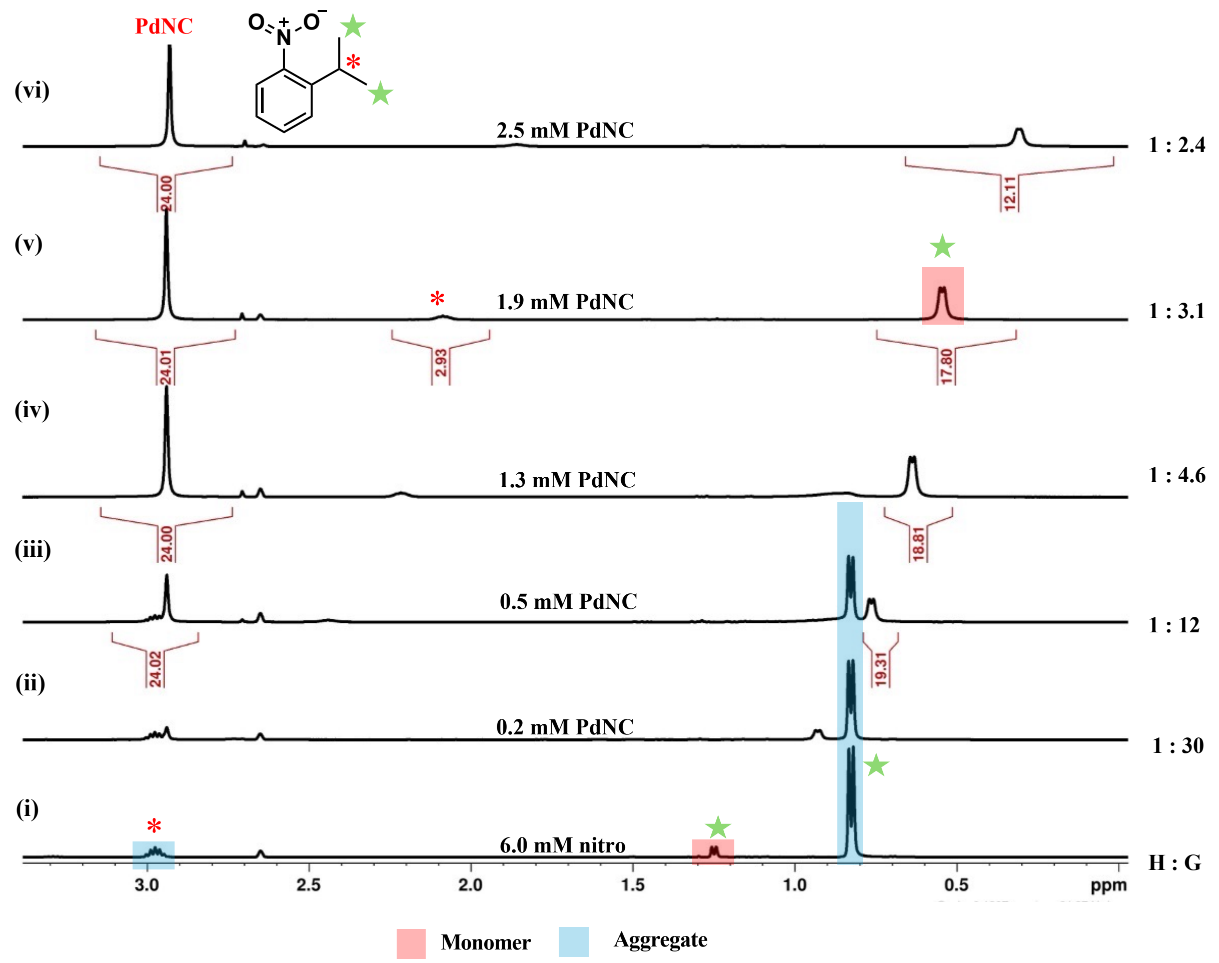

Figure S2: ^{1}H NMR partial titration spectra (500 MHz, D_2O, 25 °C) of (i) 6.0 mM *o*-nitrocumene with (ii) 0.2, (iii) 0.5, (iv) 1.3, (v) 1.9, (vi) 2.5 mM of PdNC. The green star highlights the isopropyl protons of monomer (red) and aggregates (violet), respectively. The proton integration values with respect to PdNC represented by red labels under the peaks, reveal that a maximum of 3 molecules is encapsulated within the PdNC.

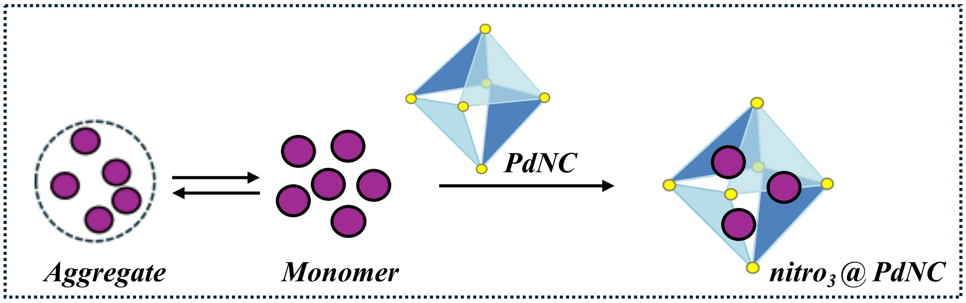

Encapsulation of *o*-nitrocumene with PdNC (Host region)

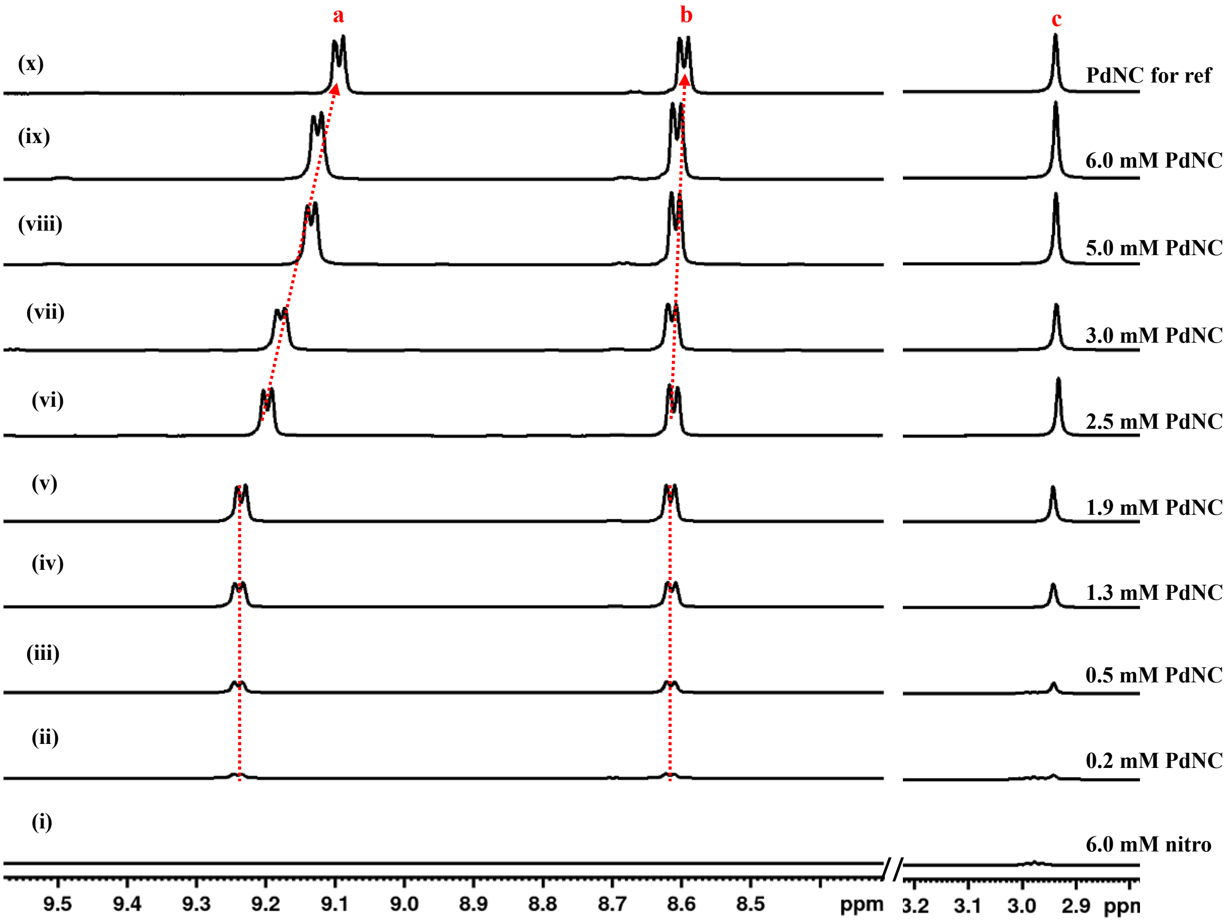

Figure S3: ^{1}H NMR partial titration spectra (500 MHz, D_2O, 25 °C) revealing the host region during the addition of PdNC to 6.0 mM *o*-nitrocumene. Both PdNC ‘a’ and ‘b’ protons remain at a constant chemical shift up to a host:guest ratio of approximately 1:3. Subsequent addition of PdNC results in a shift of these peaks towards the uncomplexed host region, providing further confirmation of three *o*-nitrocumene molecules occupying each PdNC cavity.

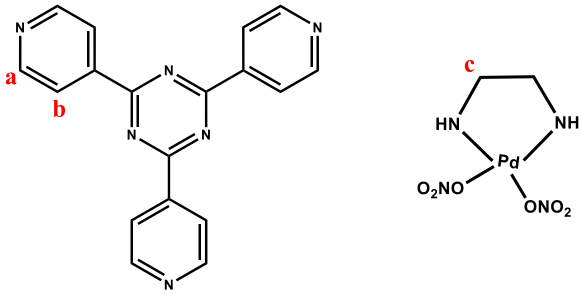

^{1}H NMR titration spectra of *o*-NC with PdNC (host → guest) (Host region)

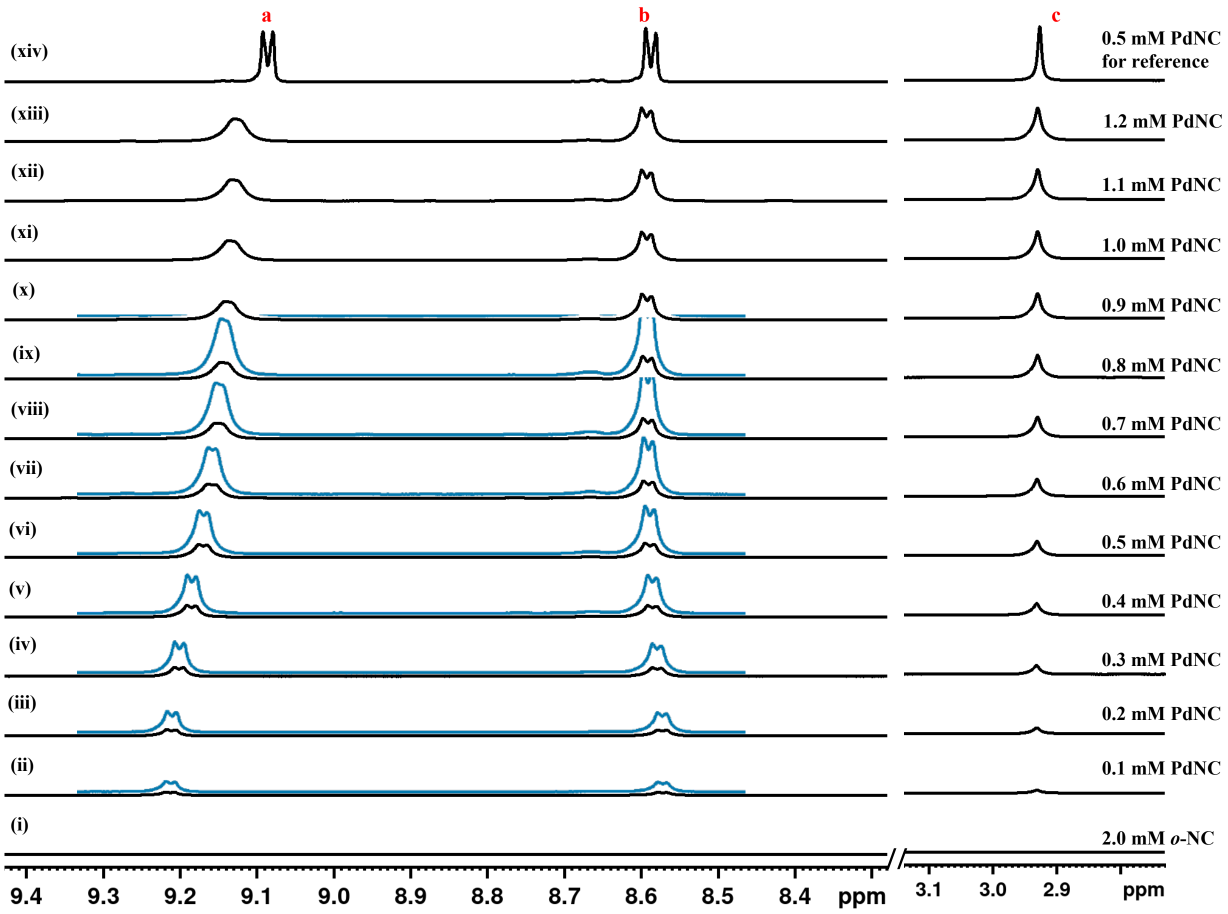

Figure S4: ^{1}H NMR titration spectra (500 MHz, D_2O, 25 °C) of (i) 2.0 mM *o*-NC with (ii) 0.1, (iii) 0.2, (iv) 0.3, (v) 0.4, (vi) 0.5, (vii) 0.6, (viii) 0.7, (ix) 0.8, (x) 0.9, (xi) 1.0, (xii) 1.1 and (xiii) 1.2 mM PdNC. 0.5 mM PdNC spectrum (xiv) is given for reference.

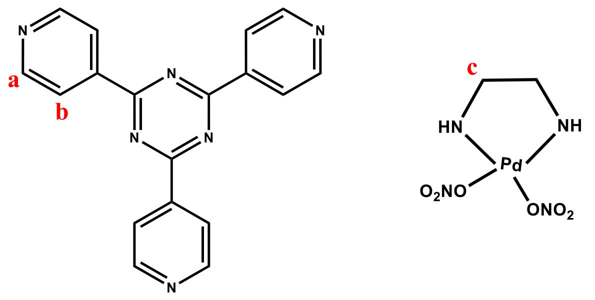

A plot of chemical shift of complexed guest vs PdNC concentration

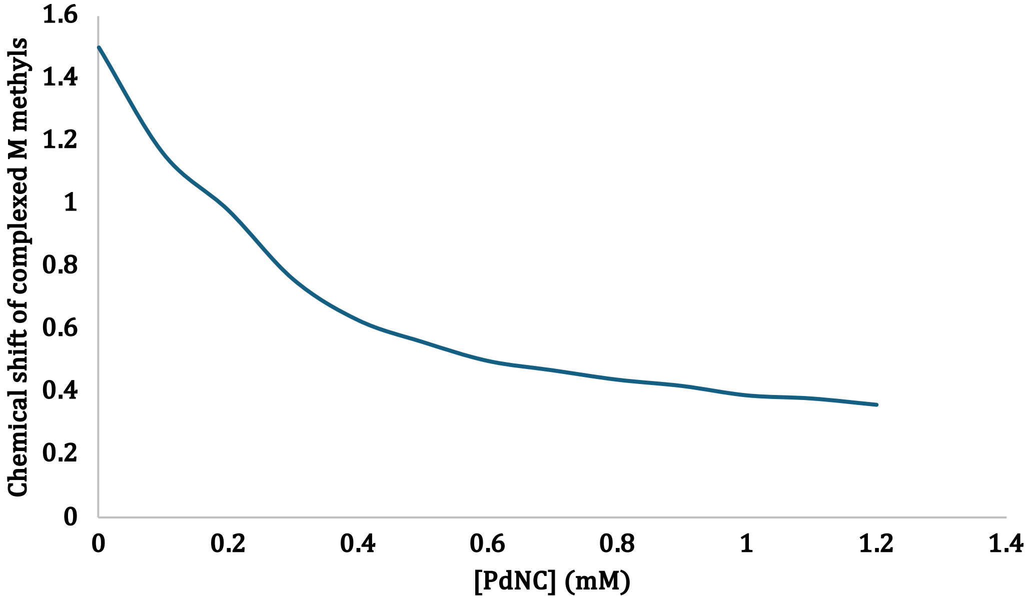

Figure S5: Dependence of the chemical shift of complexed monomer *i*-Pr methyl groups on PdNC concentration. The chemical shift of the complexed methyl protons decreases markedly at low [PdNC], then shows only slight changes once the [PdNC] reaches approximately 0.7-1.2 mM, where the curve begins to level off. This behavior, with the chemical shift decreasing from about 1.6 to 0.4 ppm as [PdNC] increases from 0 to 1.4 mM, is consistent with the formation of a 1:3 host:guest complex.

__^{1}H NMR titration spectra of *o*-NC with PdNC (guest → host) (host region)__

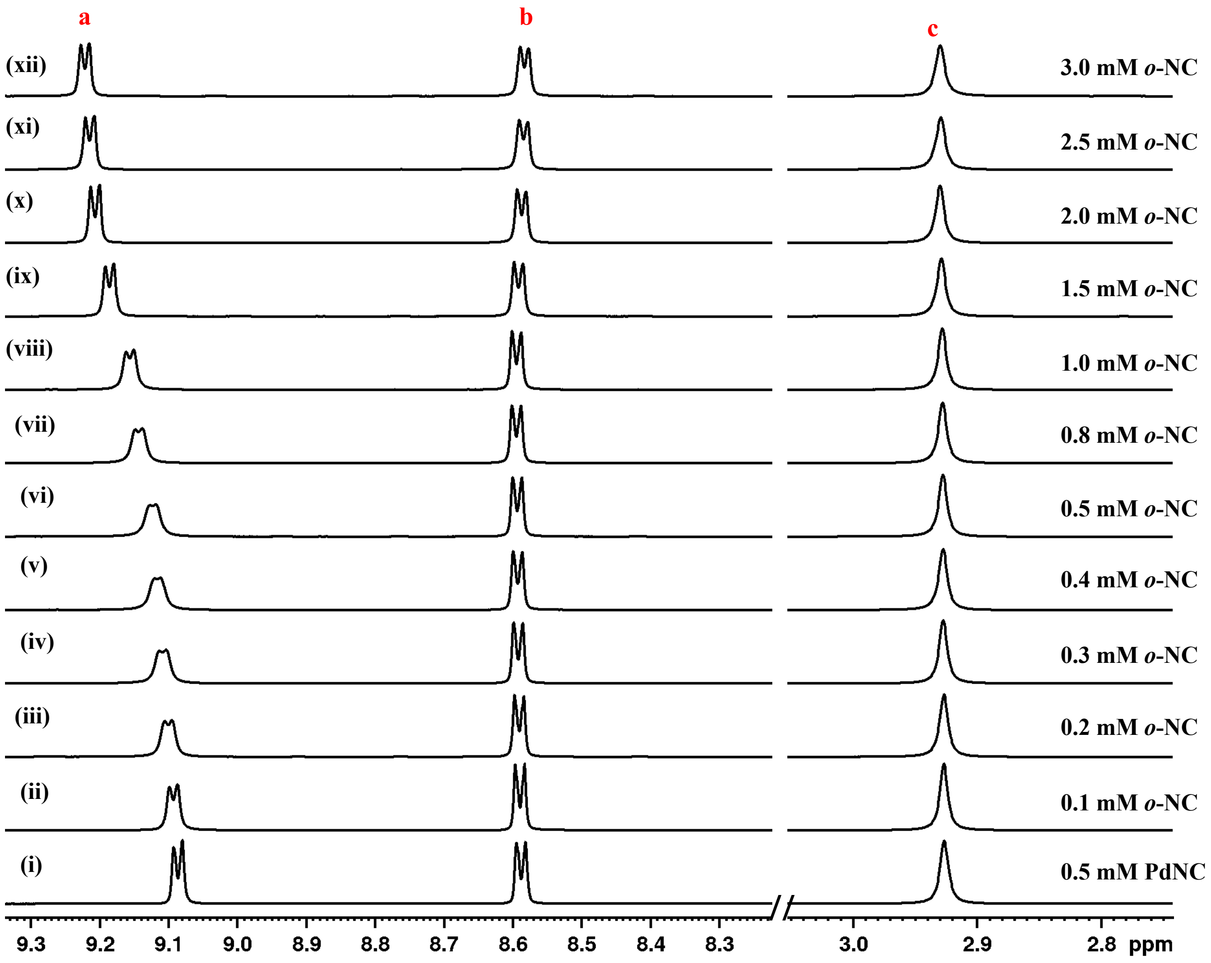

Figure S6: ^{1}H NMR partial titration spectra (500 MHz, D_2O, 25 °C) showing the host region of (i) 0.5 mM PNC with (ii) 0.1, (iii) 0.2, (iv) 0.3, (v) 0.4, (vi) 0.5, (vii) 0.8, (viii) 1.0, (ix) 1.5, (x) 2.0, (xi) 2.5 and (xii) 3.0 mM *o*-NC. The 'a' proton signal on the Pd cage progressively shifts towards a more de-shielded chemical environment, while 'b' proton exhibits slight shielding.

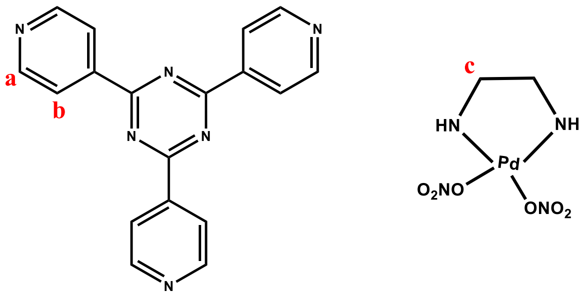

Variable Temperature ^{1}H NMR spectra of *o*-NC@PdNC complex (isopropyl region)

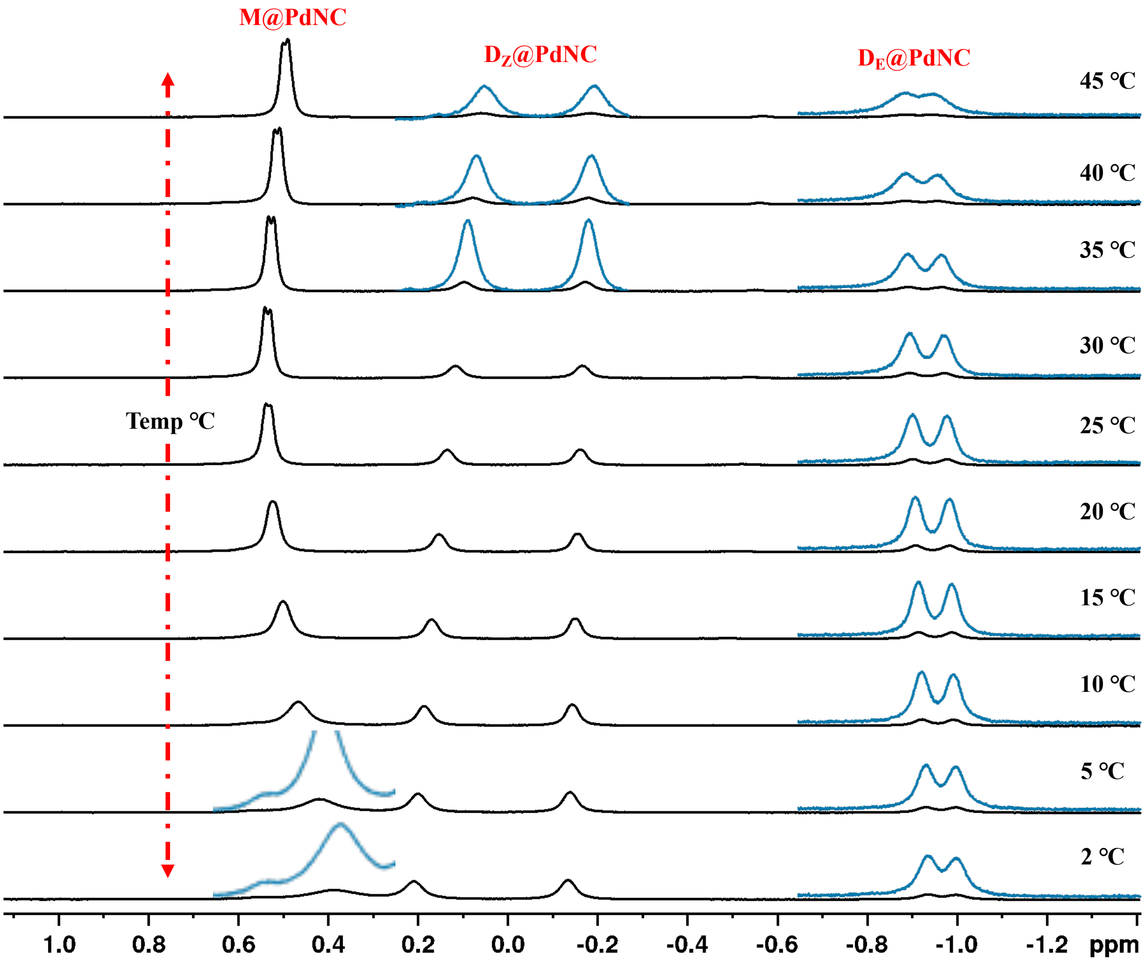

Figure S7: Variable temperature (2 – 45 °C) ^{1}H NMR (500 MHz, D_2O) spectra of 0.7 mM PdNC complexed 2.0 mM *o*-NC protons. Blue line represents the blowup of specific regions. Monomer signals broaden with decreasing temperature, while dimer signals become sharper at higher temperatures. A small shoulder peak observed to the left of broad M signal at 2 °C arises from the -CH- protons of the *i*-Pr methyl groups within the D_Z molecule.

Table S1: NMR integral values of complexed monomer (M), dimers (D_Z, D_E) with respect to PdNC is tabulated. The total integral value remains nearly constant, reinforcing the idea that the monomer and dimer within the cage are interconverted in response to changes in temperature

T °C	M	D_Z	D_E	total
**2**	5.73	9.69	2.12	17.54
**5**	6.53	8.97	2.18	17.68
**10**	7.54	7.93	2.25	17.72
**15**	8.28	7.00	2.25	17.53
**20**	9.07	6.12	2.20	17.39
**25**	9.69	5.24	2.20	17.13
**30**	10.12	4.46	1.95	16.53
**35**	10.49	3.78	1.76	16.03
**40**	10.90	3.11	1.59	15.6
**45**	11.08	2.58	1.36	15.02

<u>**Variable Temperature ^{1}H NMR spectra of *o*-NC@PdNC complex (host region)**</u>

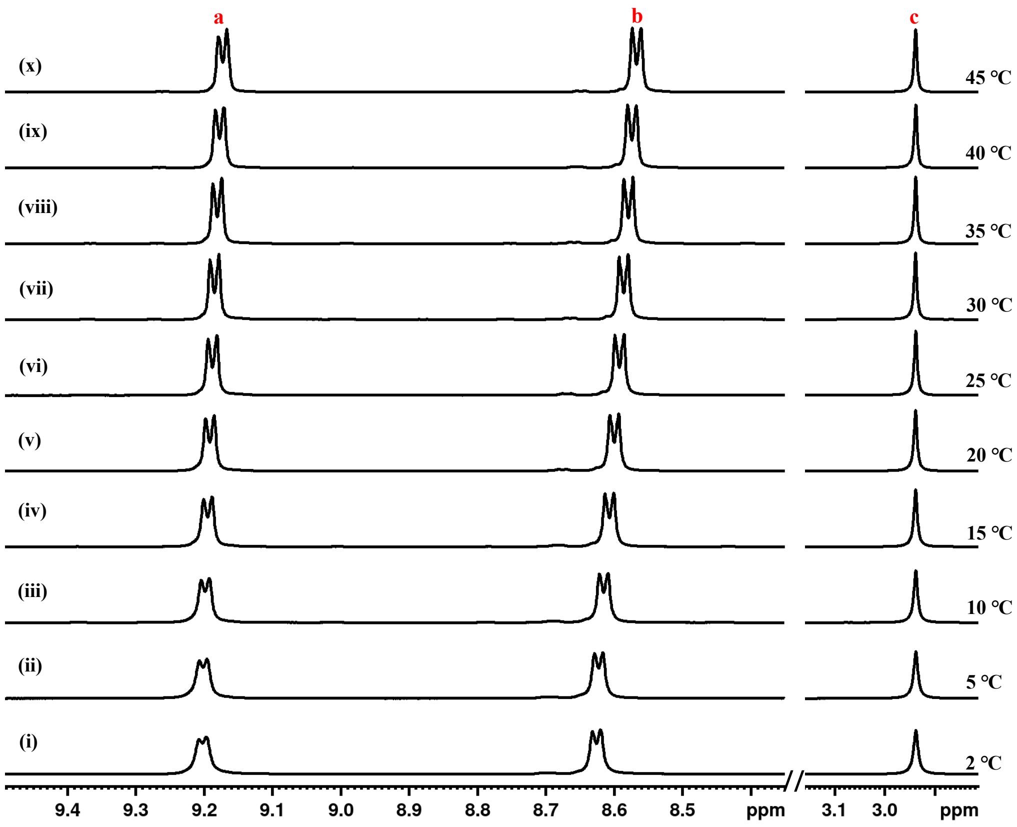

Figure S8: Variable temperature (2 – 45 °C) ^{1}H NMR (500 MHz, D_2O) spectra of illustrating the host region of the 2.0 mM*o*-NC@0.7mMPdNC complex. At lower temperatures, host 'a' proton appeared broad probably due to restricted exchange accompanied by the presence of three different cage environments.

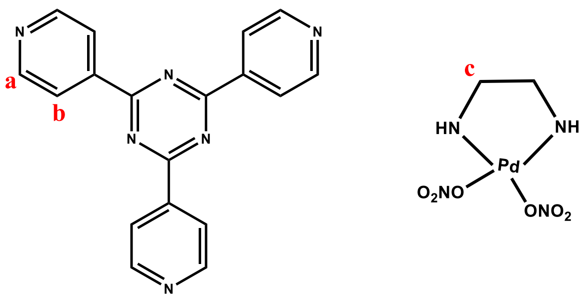

$^{13}C\{^1H\}$ NMR spectrum of *o*-NC@PdNC complex (full region)

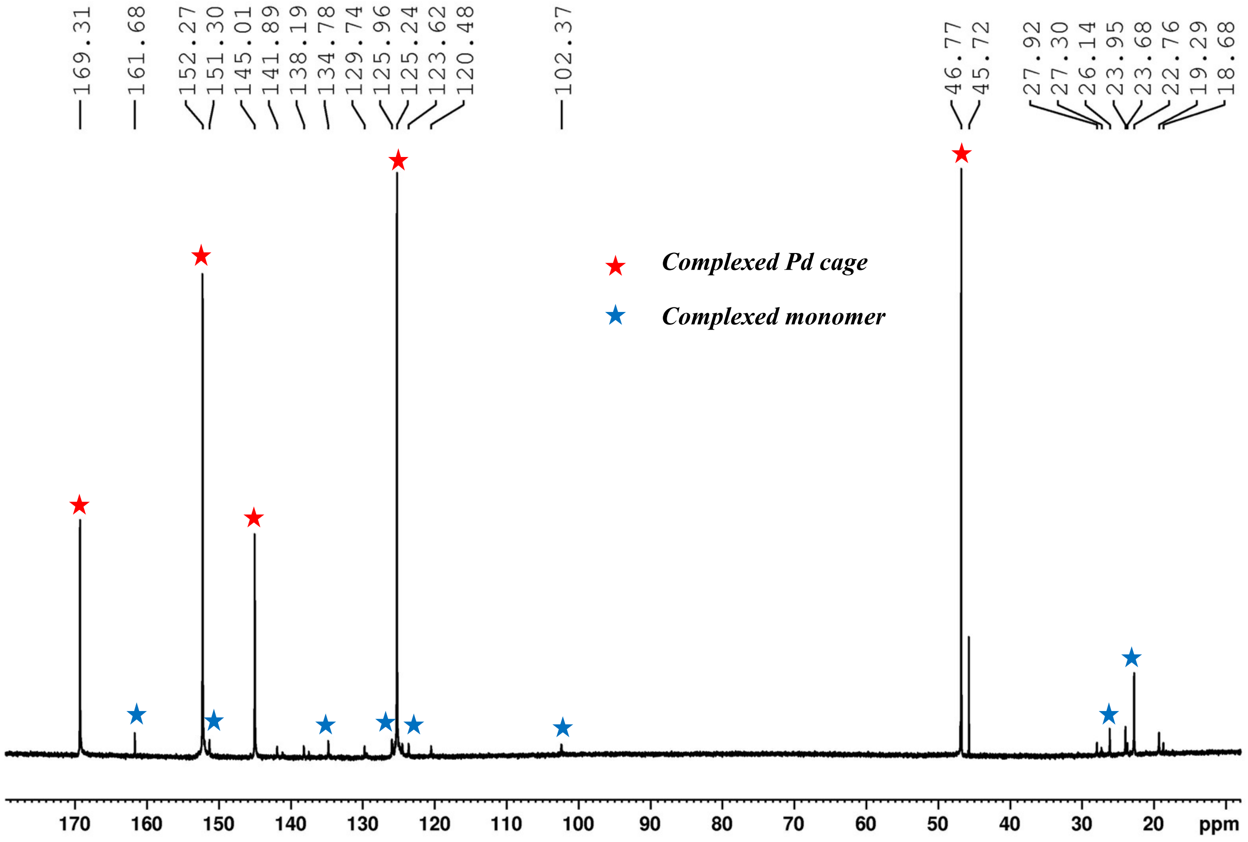

Figure S9: $^{13}C\{^1H\}$ NMR spectrum (200 MHz, D_2O, 25 °C) of 5.0 mM PdNC complexed with 17.0 mM *o*-NC guest. Complexed PdNC signals are labelled by red stars and that of complexed guest are blue stars.

$^{13}C\{^{1}H\}$ NMR spectrum of *o*-NC@PdNC complex (guest aliphatic region)

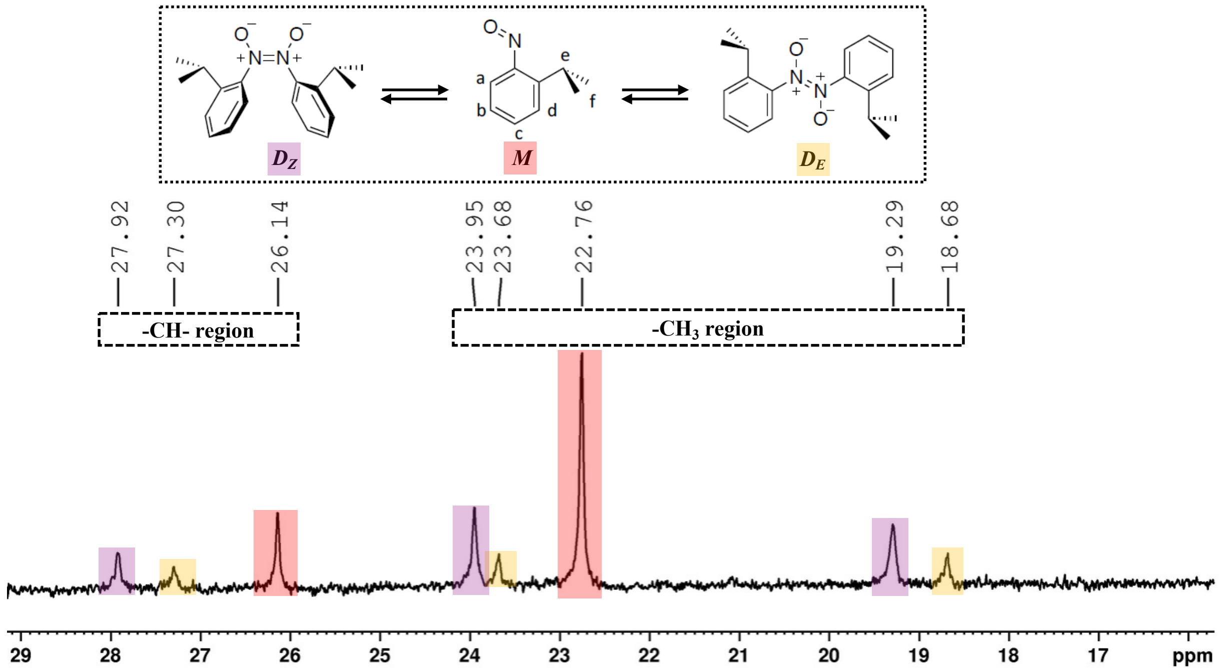

Figure S10: $^{13}C\{^{1}H\}$ NMR partial spectrum (200 MHz, D_2O, 25 °C) of 5.0 mM PdNC complexed with 17.0 mM of guest exhibiting –CH- and $–CH_3$ signals of complexed M, D_Z and D_E.

Table S2: Chemical shifts of monomer and dimer species in $CDCl_3$ as well as inside the PdNC are tabulated for comparison. Presence of five distinct isopropyl signals further ensures the presence of complexed monomers and two different dimers.

Free species	Chemical shift in $CDCl_3$ (ppm)		Chemical shift in PdNC (ppm)	
	-CH-	$-CH_3$	-CH-	$-CH_3$
**M**	27.3	24.4	26.1	22.7
**D_Z**	29.1	25.8, 21.9	27.9	23.9, 19.3
**D_E**	29.4	23.4	27.3	23.6, 18.6

^{1}H - ^{13}C HSQC spectrum of PdNC complexed *o*-NC (guest aliphatic region)

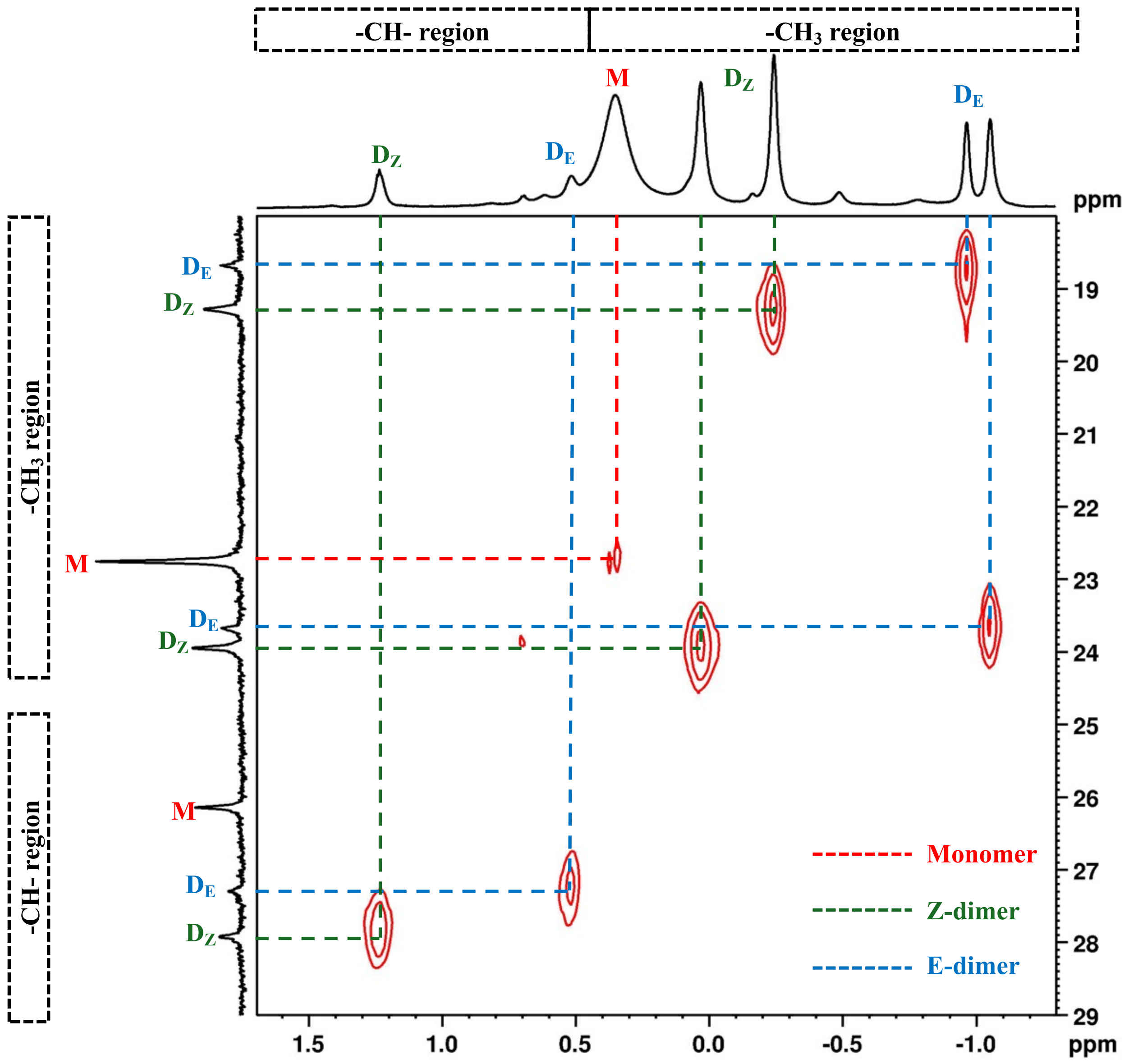

Figure S11: Expanded view of the aliphatic region of the ^{1}H- ^{13}C HSQC spectrum (800 MHz, D_2O, 25 °C) of 5.0 mM PdNC complexed with 17.0 mM guest. Cross peaks correspond to through bond correlations between complexed guest protons (horizontal axis) and their directly attached ^{13}C nuclei (vertical axis) showing correlations. Dotted lines in red, green and blue represents cross peaks of the monomer, Z-dimer and E-dimer respectively.

2D COSY spectrum of PdNC complexed *o*-NC (guest aliphatic region)

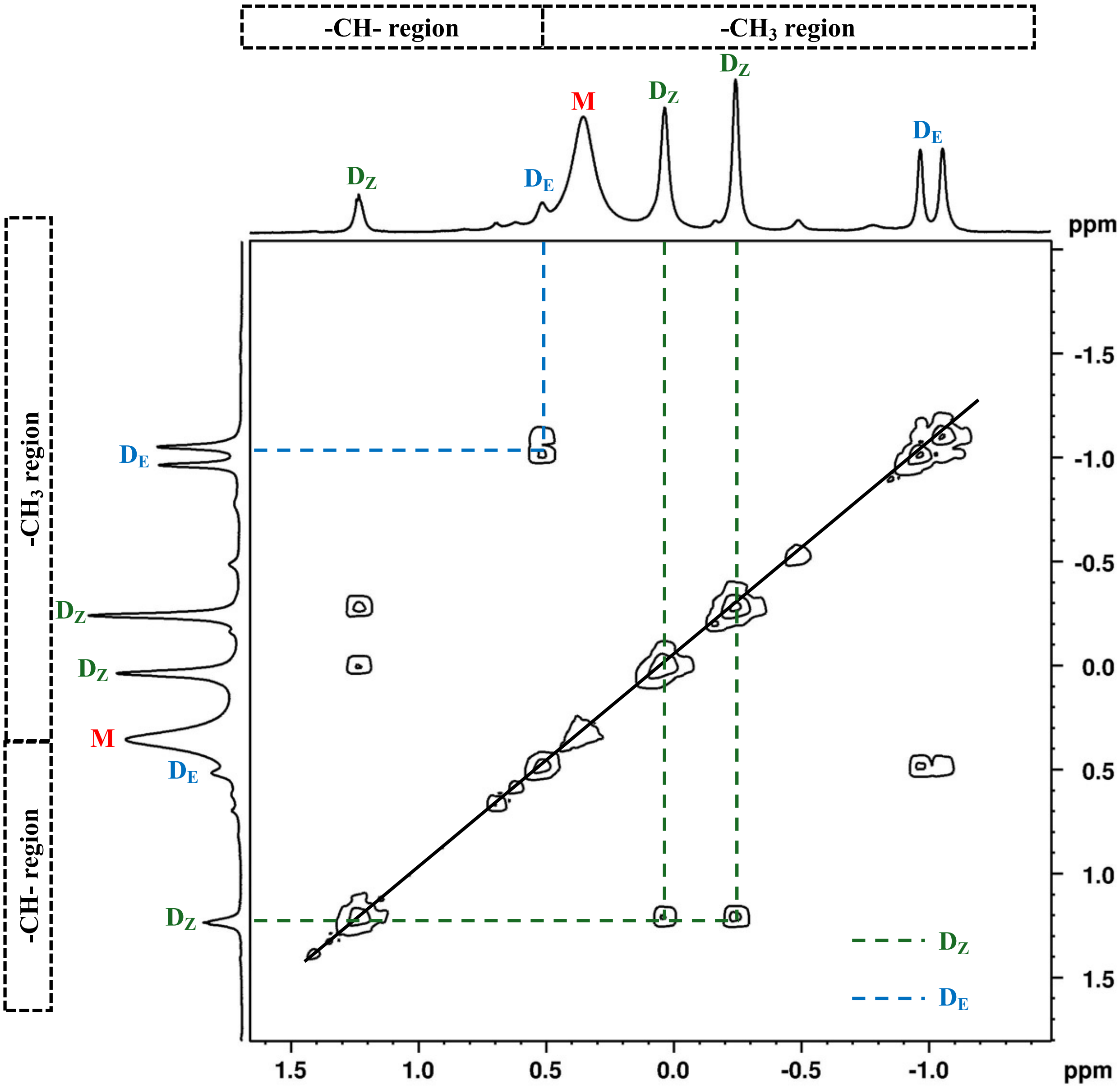

Figure S12: The 2D COSY spectrum (800 MHz, D_2O, 25 °C) of a solution containing 15.0 mM *o*-NC + 5.0 mM PdNC reveals correlation between complexed guest aliphatic protons, providing insights into the environment of the isopropyl groups. The presence of only one set of cross peaks indicates that the two isopropyl protons experience a similar environment when encapsulated within the Pd cage.

Concentration based titration of *o*-NC in water (guest isopropyl region)

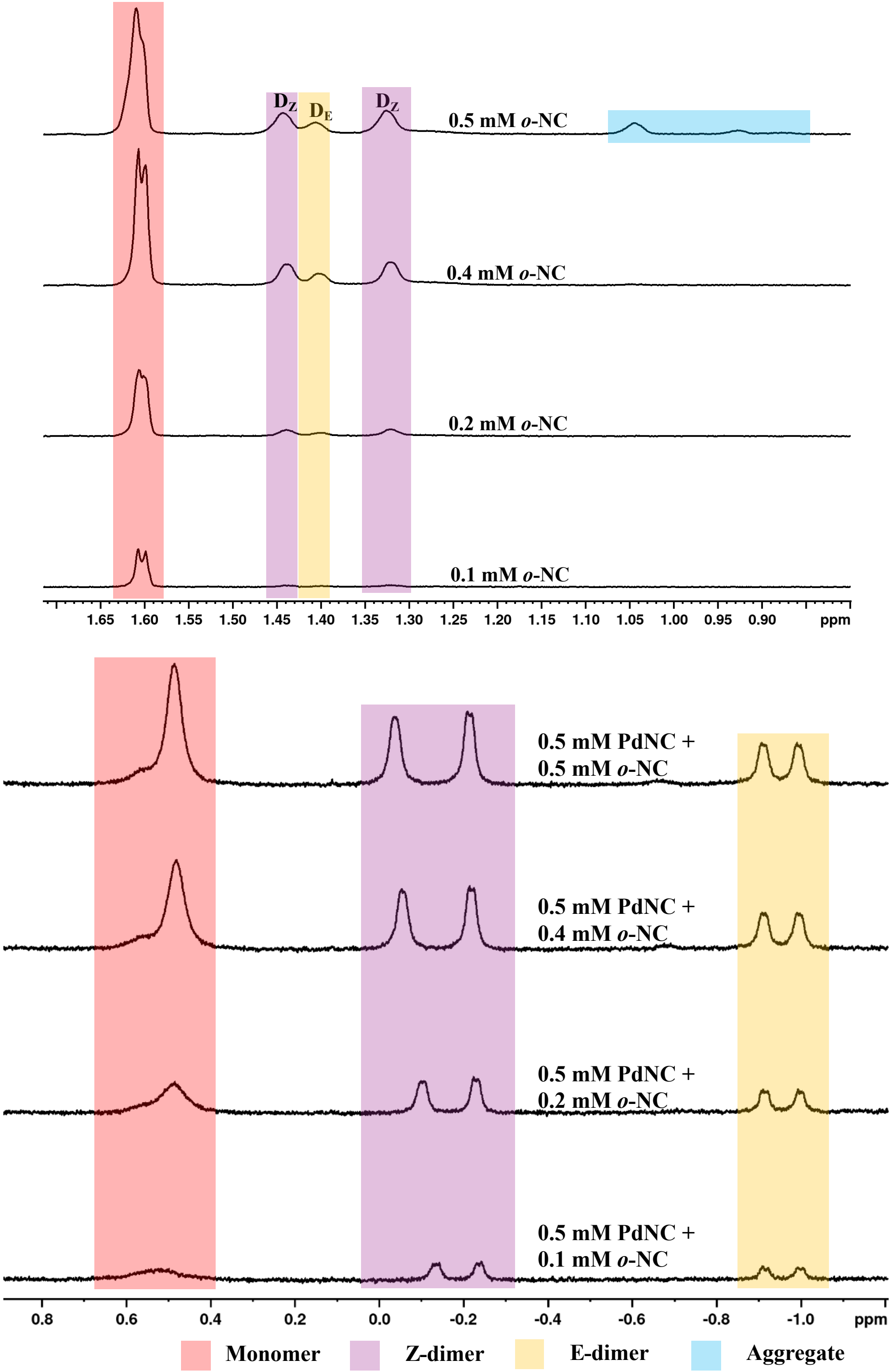

Figure S13:^{1}H NMR spectra (500 MHz, D_2O, 25 °C) of *o*-NC at different concentrations in water (top spectrum) vs in PdNC (bottom spectrum).

Table S3: Chemical shift difference between isopropyl methyls of dimers expressed in ppm

[*o*-NC] mM	**Chemical shift difference of *i*-Pr methyls in ppm**		
	**D_Z in D_2O**	**D_Z@PdNC**	**D_E@PdNC**
**0.5**	0.12	0.18	0.09
**0.4**	0.12	0.16	0.09
**0.2**	0.12	0.13	0.09
**0.1**	0.12	0.10	0.09

Table S4: Distribution of *o*-NC monomer (M) and dimer (D) species in water vs PdNC

**[*o*-NC] added (mM)**	***o*-NC distributions in water**			***o*-NC distributions in PdNC**		
	**% M**	**% D_Z**	**% D_E**	**% M**	**% D_Z**	**% D_E**
**0.1**	94.0	3.0	3.0	44.0	33.0	23.0
**0.2**	91.0	5.5	3.5	57.2	25.4	17.4
**0.4**	85.0	9.3	5.7	62.3	23.8	14.0
**0.5**	84.8	9.6	5.6	64.2	22.8	13.0

Note: The percentages shown were determined by analyzing NMR signal integrations from free monomer and dimer forms relative to the DMSO-d_6 solvent residual signal, and PdNC signals for complexed *o*-NC. The total *o*-NC concentration was normalized to the initial amount added.

^{1}H NMR spectra of adding CB8 to *o*-NC@PdNC complexed solution (guest region)

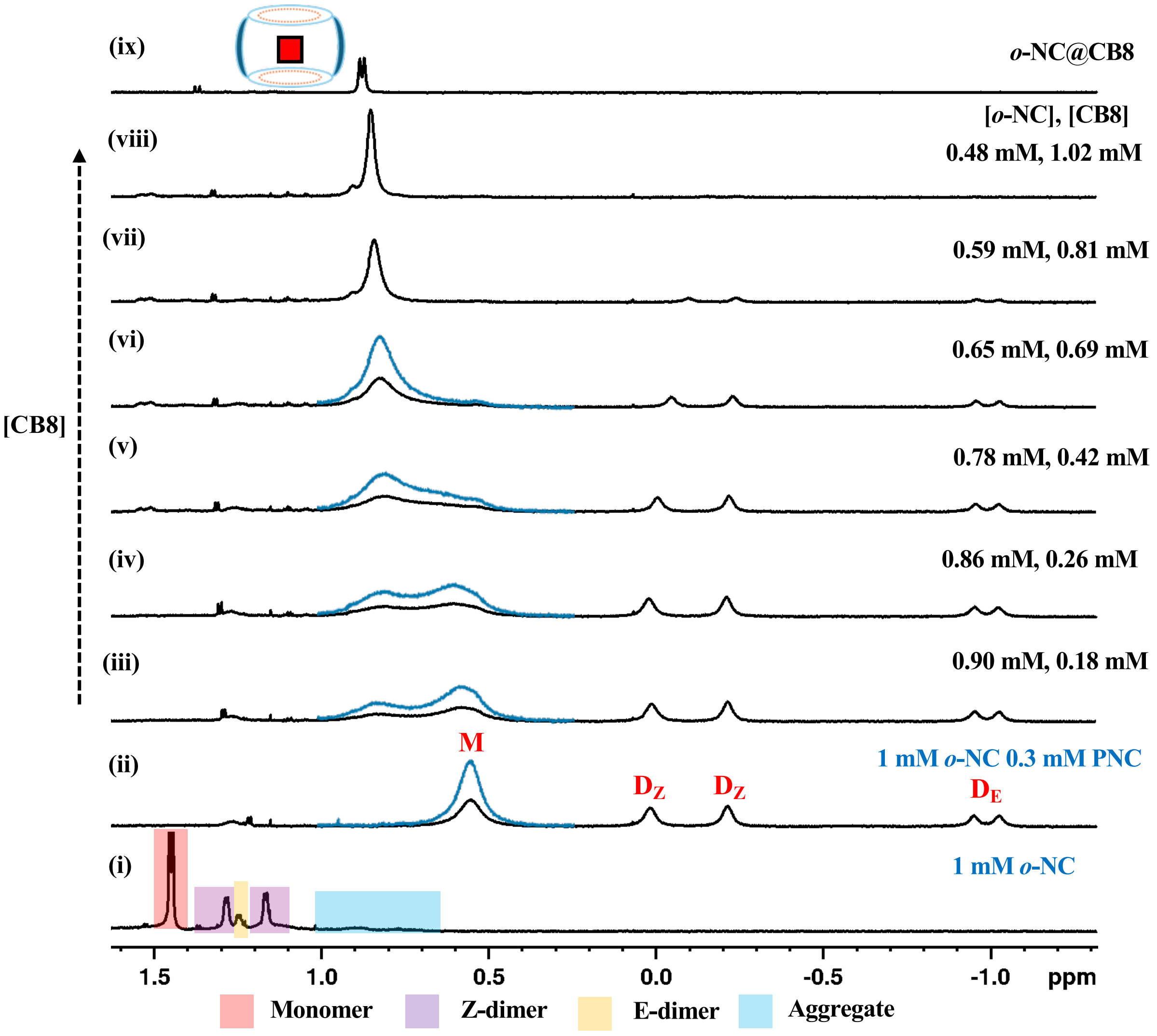

Figure S14: ^{1}H NMR partial titration spectra (800 MHz, D_2O, 25 °C) of (i) *o*-NC, (ii) *o*-NC@PdNC with (iii) – (ix) increasing addition of CB8. *o*-NC@CB8 complexed spectrum (viii) is given for reference. The blue line spectrum highlights specific regions in detail. Initial addition of CB8 results in broadening and a down-field shift of first complexed peak, confirming the presence of monomers. Subsequent additions lead to the complete displacement of dimers from the Pd cage, forming M@CB8 complex.

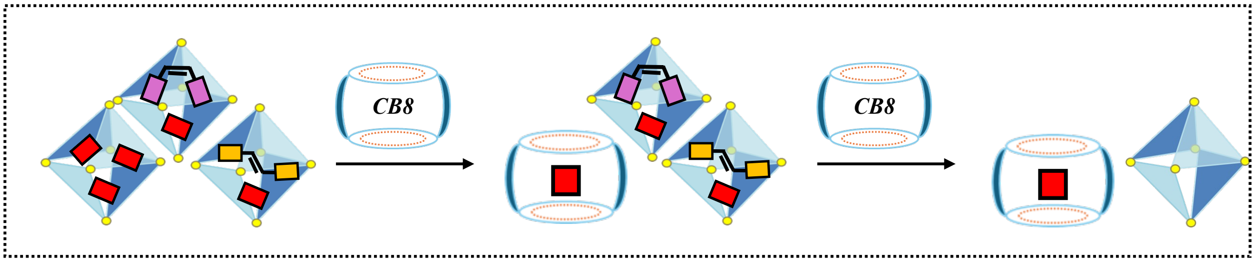

2D DOSY spectrum of free PdNC and complexed PdNC with *o*-NC in aqueous solution

1. **Free PdNC**

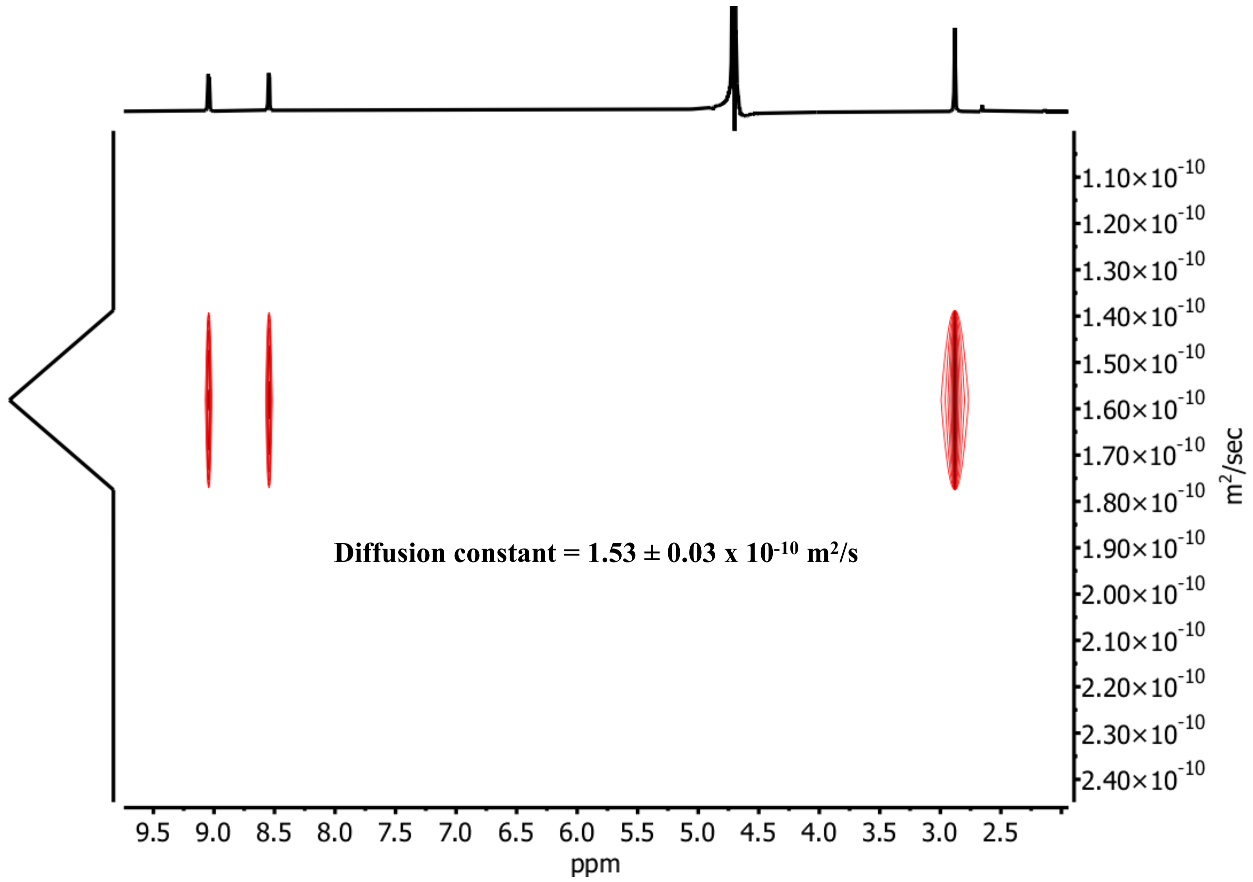

Figure S15: 2D DOSY NMR spectrum (800 MHz, D_2O, 25 °C) of free 0.5 mM Pd nanocage. An average diffusion constant (D) of 1.53 ± 0.03 x 10^{-10} m^2/s is observed for free Pd nanocage protons.

2. 2 mM *o*-NC with 0.7 mM PdNC

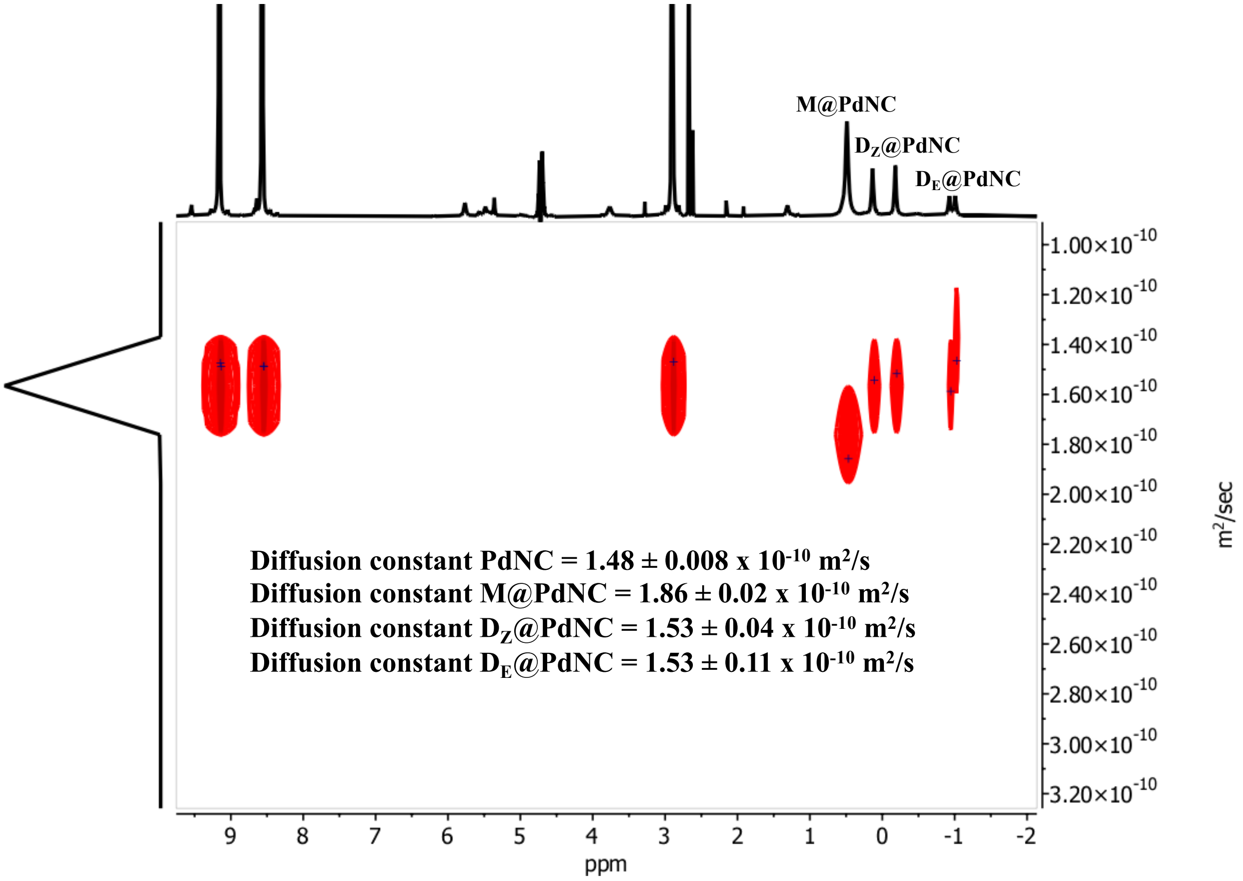

Figure S16: 2D DOSY NMR spectrum (800 MHz, D_2O, 25 °C) of 2 mM *o*-NC and 0.7 mM PdNC complex reveals a decrease in the PdNC diffusion coefficient from 1.53 to 1.48 x 10^{-10} m^2/s, confirming complex formation. The dimeric complexes diffuse at similar rates (1.53 x 10^{-10} m^2/s), whereas the monomer exhibits faster diffusion (1.48 x 10^{-10} m^2/s), suggesting a possible exchange of monomers between the cage and solution.

2D NOESY spectrum of *o*-NC at different mixing times at 25 °C (5, 10, 20, 50, 100, 200, 300, 400, 500, 600 & 700 ms)

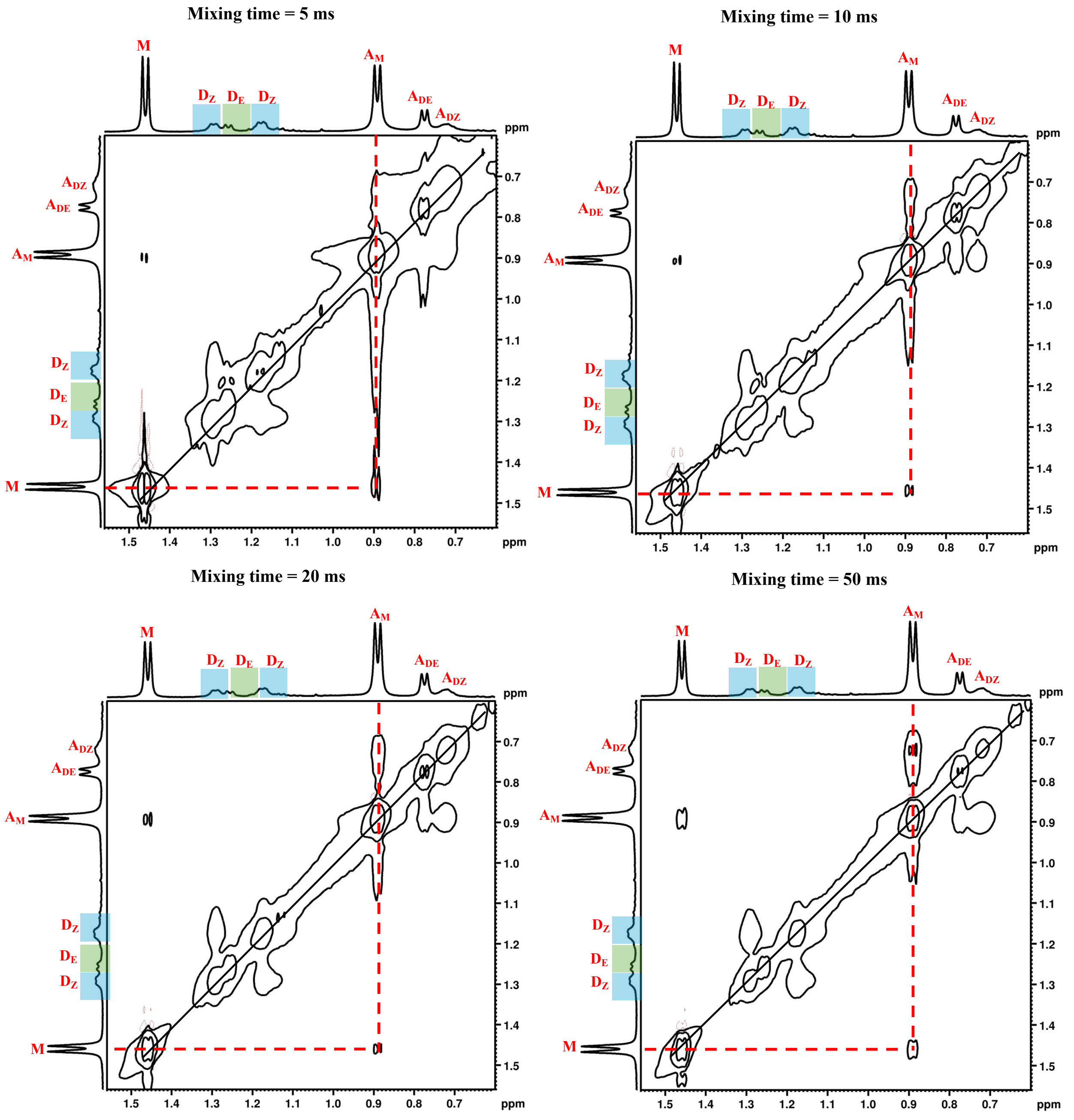

Figure S17: 2D NOESY (500 MHz, D_2O, 25 °C) of 2.2 mM *o*-NC acquired at 5 - 50 ms mixing time reveal exchange correlations between isopropyl groups in water. The observed cross-peaks indicate dynamic exchange between monomeric species in solution and aggregated forms.

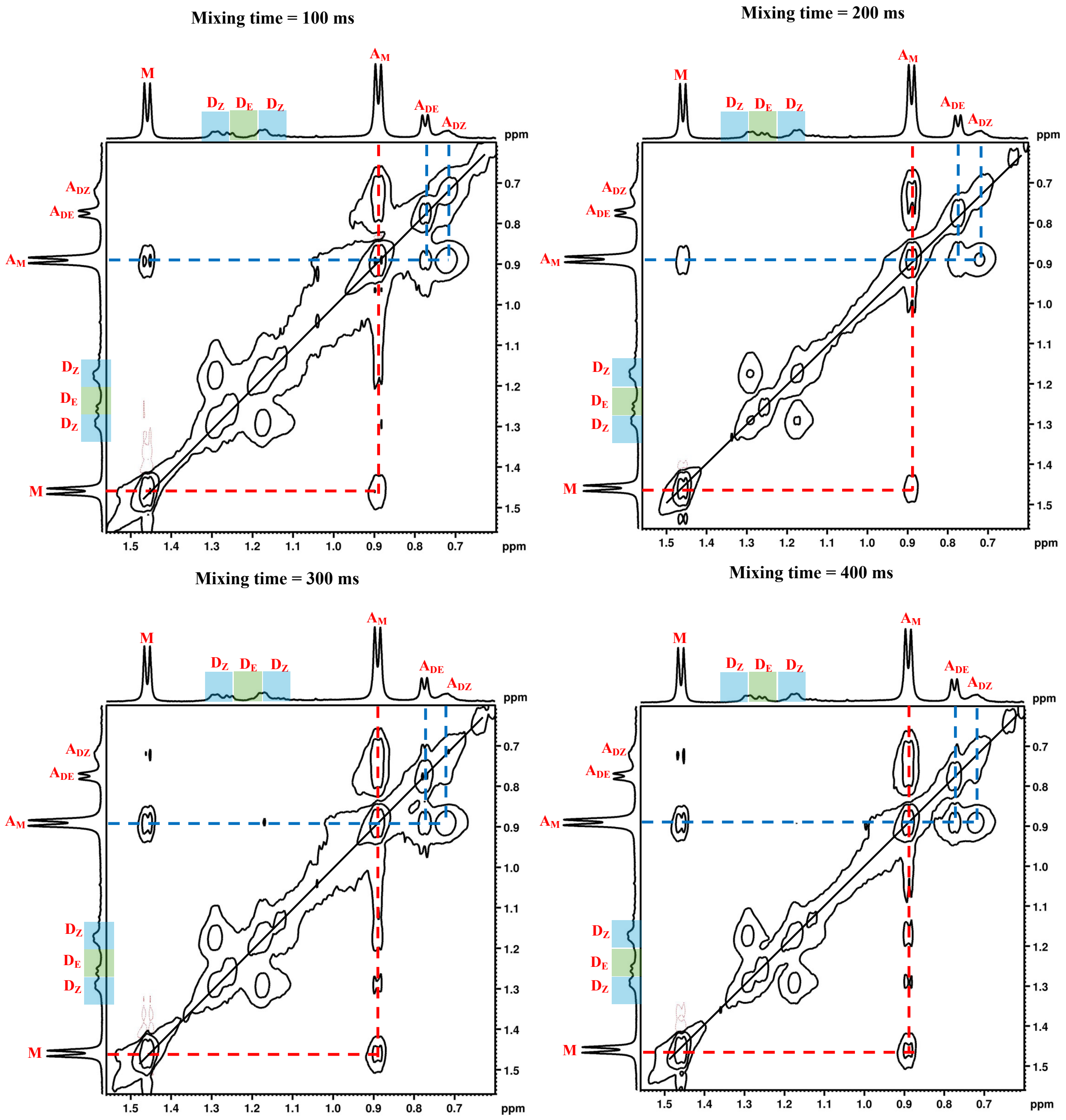

Figure S18: 2D NOESY (500 MHz, D_2O, 25 °C) of 2.2 mM *o*-NC acquired at 100 - 400 ms mixing reveal exchange correlations between isopropyl groups in water. The observed cross-peaks highlighted by red lines represent dynamic exchange between monomeric species in solution and aggregated forms, while those indicated by blue lines reflect exchange among different aggregate species.

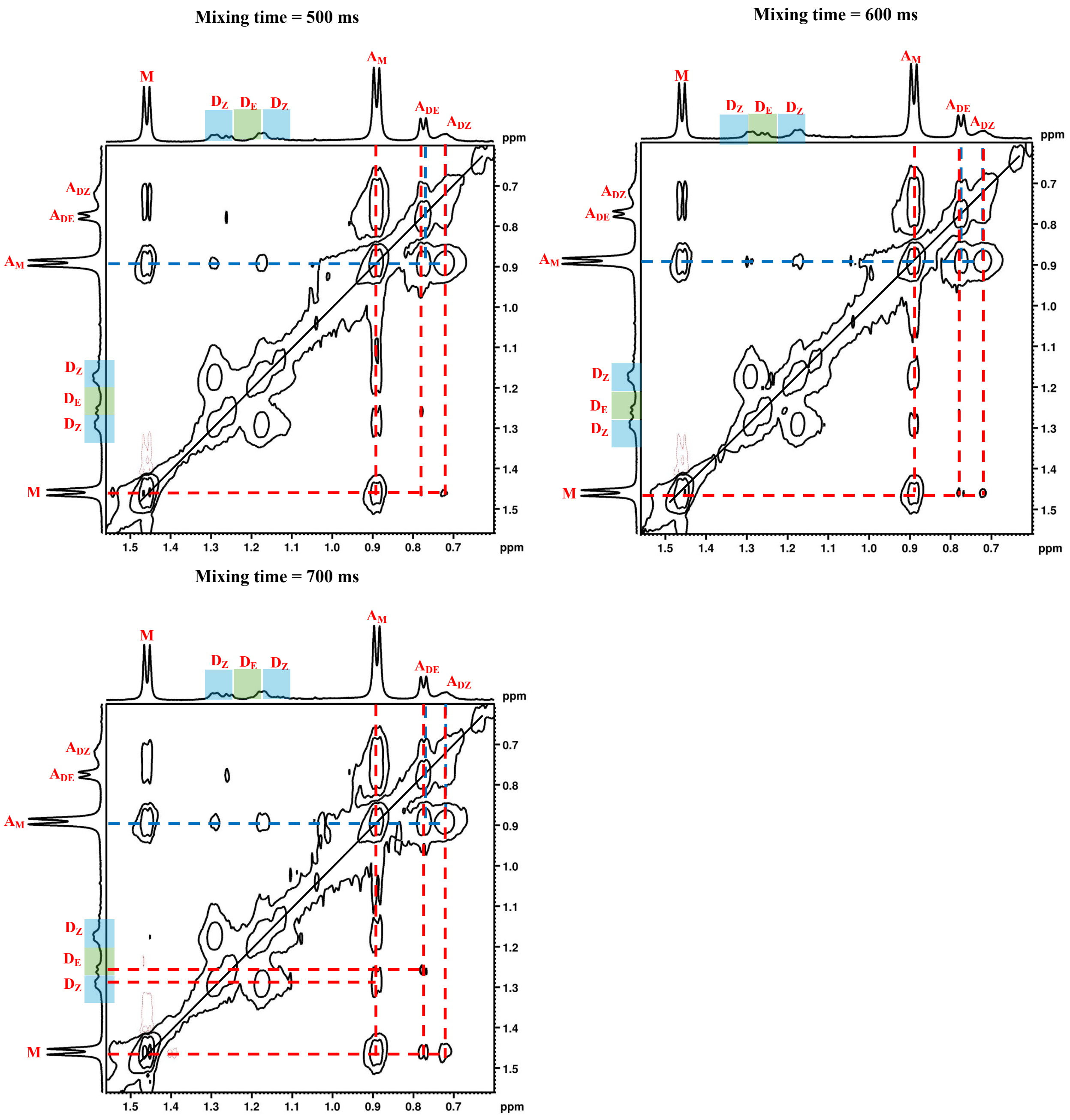

Figure S19: 2D NOESY (500 MHz, D_2O, 25 °C) of 2.2 mM *o*-NC acquired at 500 - 700 ms mixing reveal exchange correlations between isopropyl groups in water. The observed cross-peaks highlighted by red lines represent dynamic exchange between monomeric species in solution and aggregated forms, while those indicated by blue lines reflect exchange among different aggregate species.

2D NOESY spectrum of *o*-NC@PdNC at different mixing times at 25 °C (10, 30, 50, 100, 200, 300, 400, 500 & 600 ms)

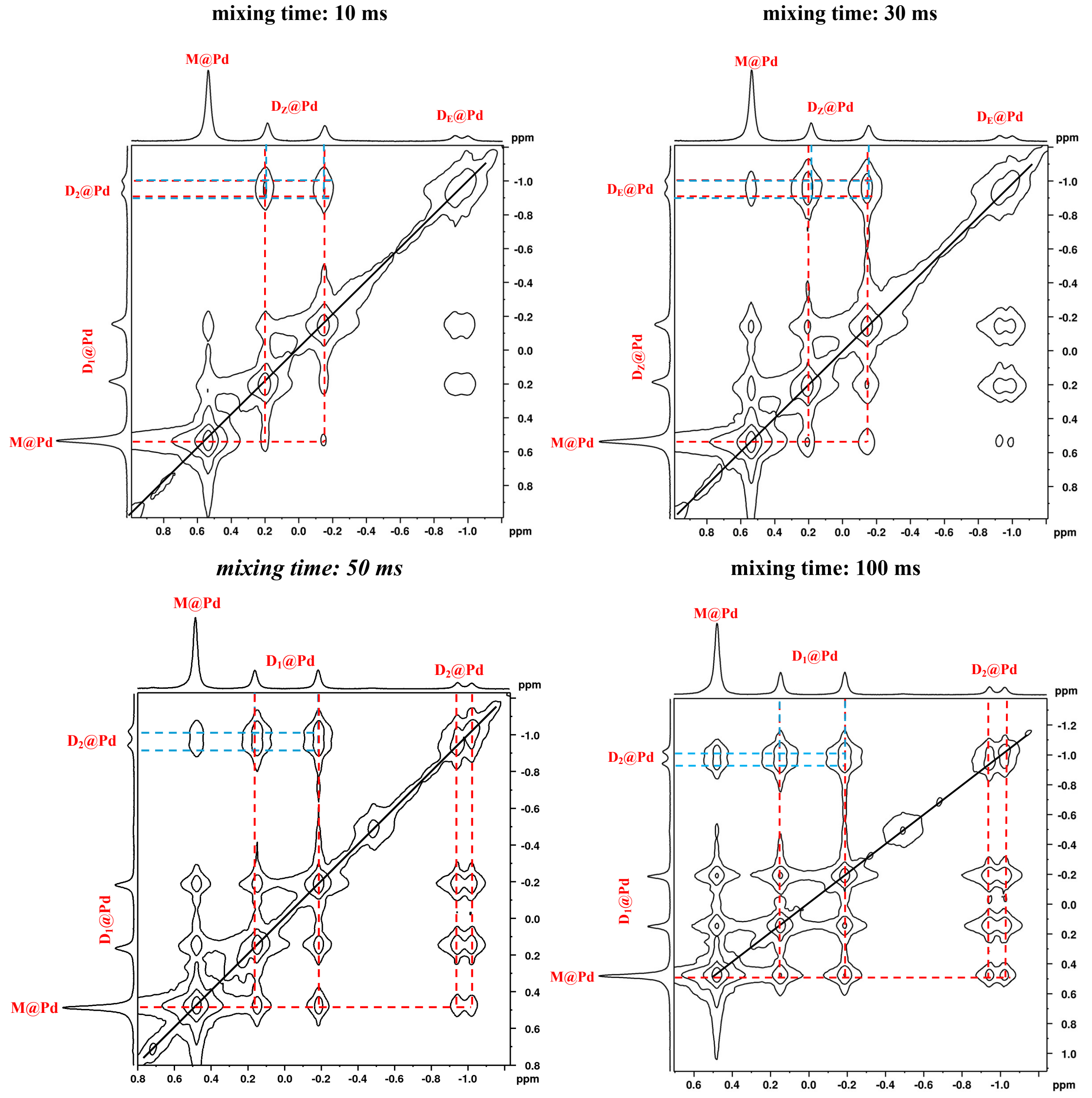

Figure S20: 2D NOESY (800 MHz, D_2O, 25 °C) spectra of 2.2 mM *o*-NC + 0.7 mM PdNC showing exchange correlations between complexed isopropyl groups at 10 - 100 ms mixing times. Red dotted lines indicate exchange correlations between complexed monomers and dimers, while blue dotted lines indicate exchange between D_Z and D_E.

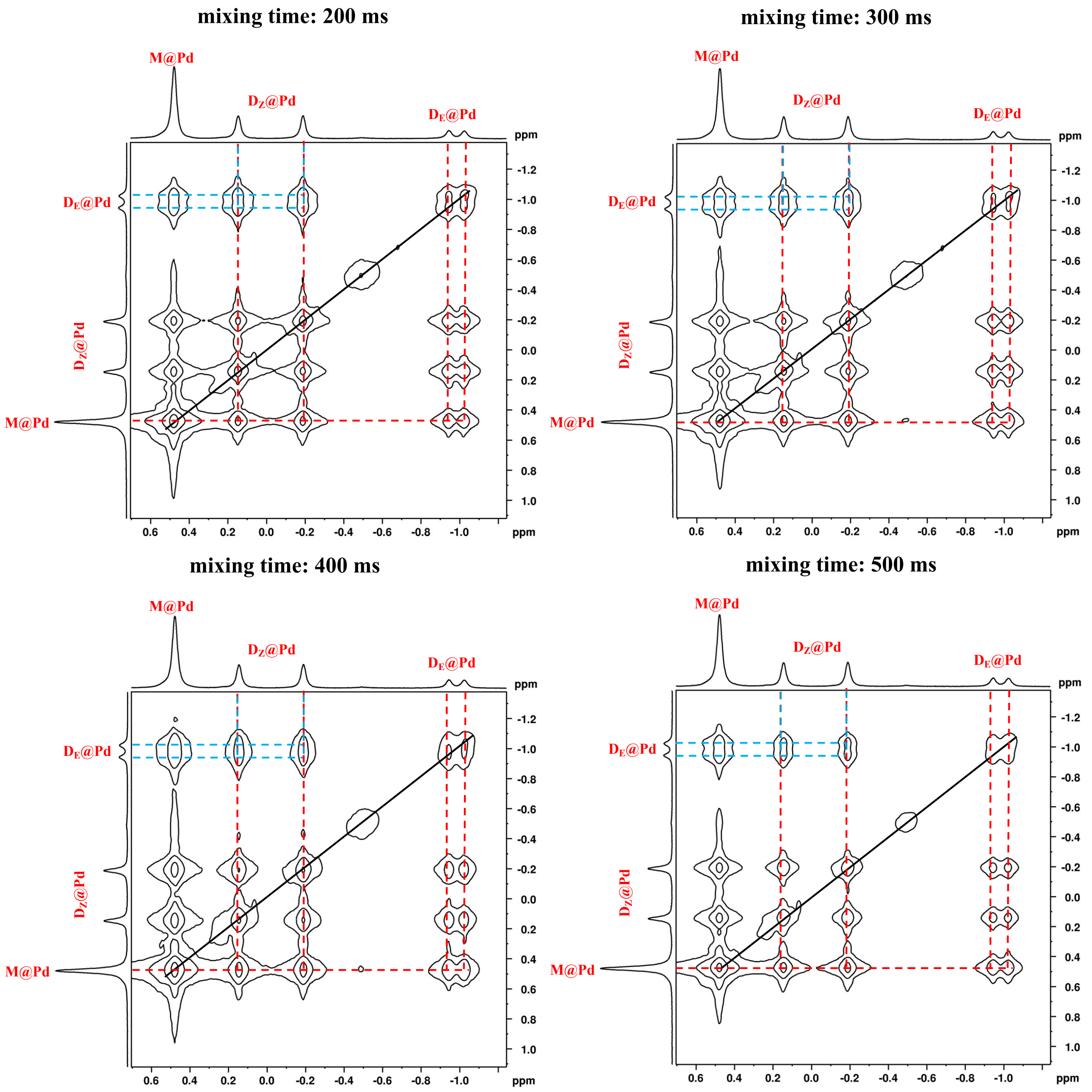

Figure S21: 2D NOESY (800 MHz, D_2O, 25 °C) spectra of 2.2 mM *o*-NC + 0.7 mM PdNC showing exchange correlations between complexed isopropyl groups at 200 - 500 ms mixing times. Red dotted lines indicate exchange correlations between complexed monomers and dimers, while blue dotted lines indicate exchange between D_Z and D_E.

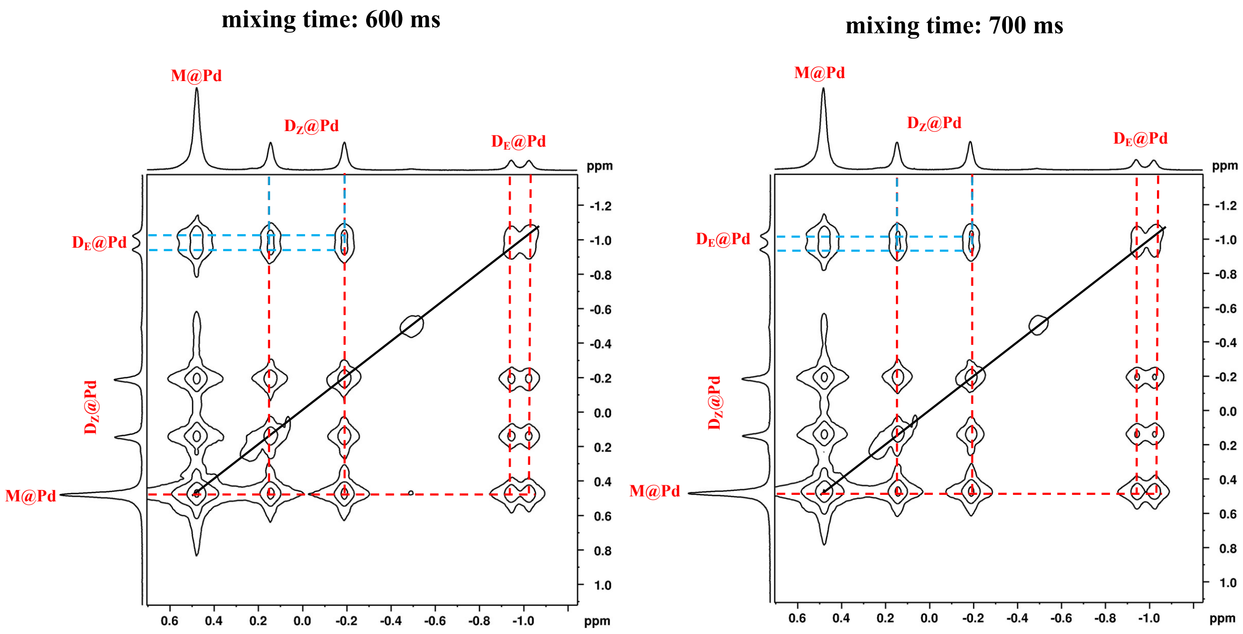

Figure S22: 2D NOESY (800 MHz, D_2O, 25 °C) spectra of 2.2 mM *o*-NC + 0.7 mM PdNC showing exchange correlations between complexed isopropyl groups at 600 - 700 ms mixing times. Red dotted lines indicate exchange correlations between complexed monomers and dimers, while blue dotted lines indicate exchange between D_Z and D_E.

Plot of 2D integral value vs exchange buildup obtained at different mixing times

Mixing time (ms)	NOE exchange integral values with respect to MeOH IS		
	D_Z- D_E	M-D_Z	M-D_E
5	0.67	0.00	0.00
10	1.24	0.34	0.00
20	1.99	0.72	0.10
50	2.49	0.85	0.21
100	2.79	1.64	0.51
200	2.01	2.22	0.82
300	1.43	2.54	0.76
400	1.06	2.25	0.78
500	0.88	2.26	0.71
600	0.59	1.99	0.66

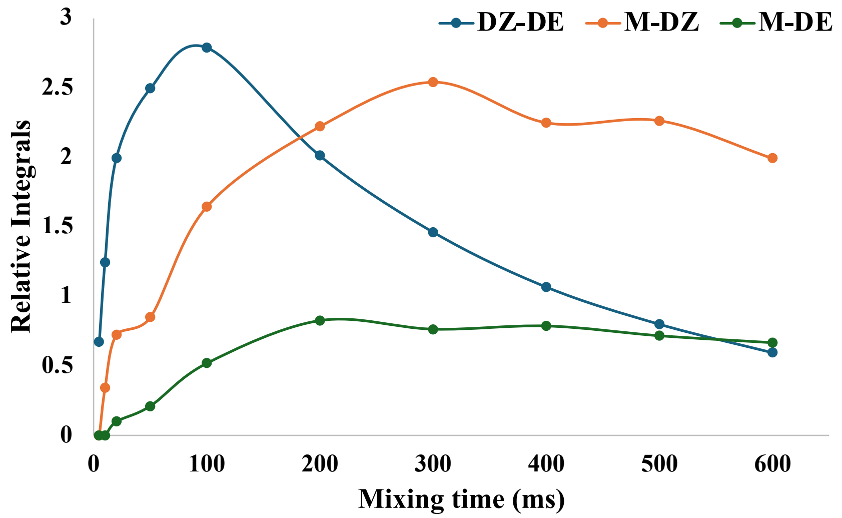

Figure S23: A plot of 2D NOE exchange integrals of D_Z-D_E, M-D_Z, M-D_E vs mixing time.

2D NOESY spectrum of *o*-NC@PdNC at different mixing times at 5 °C (10, 50, 100, & 200 ms)

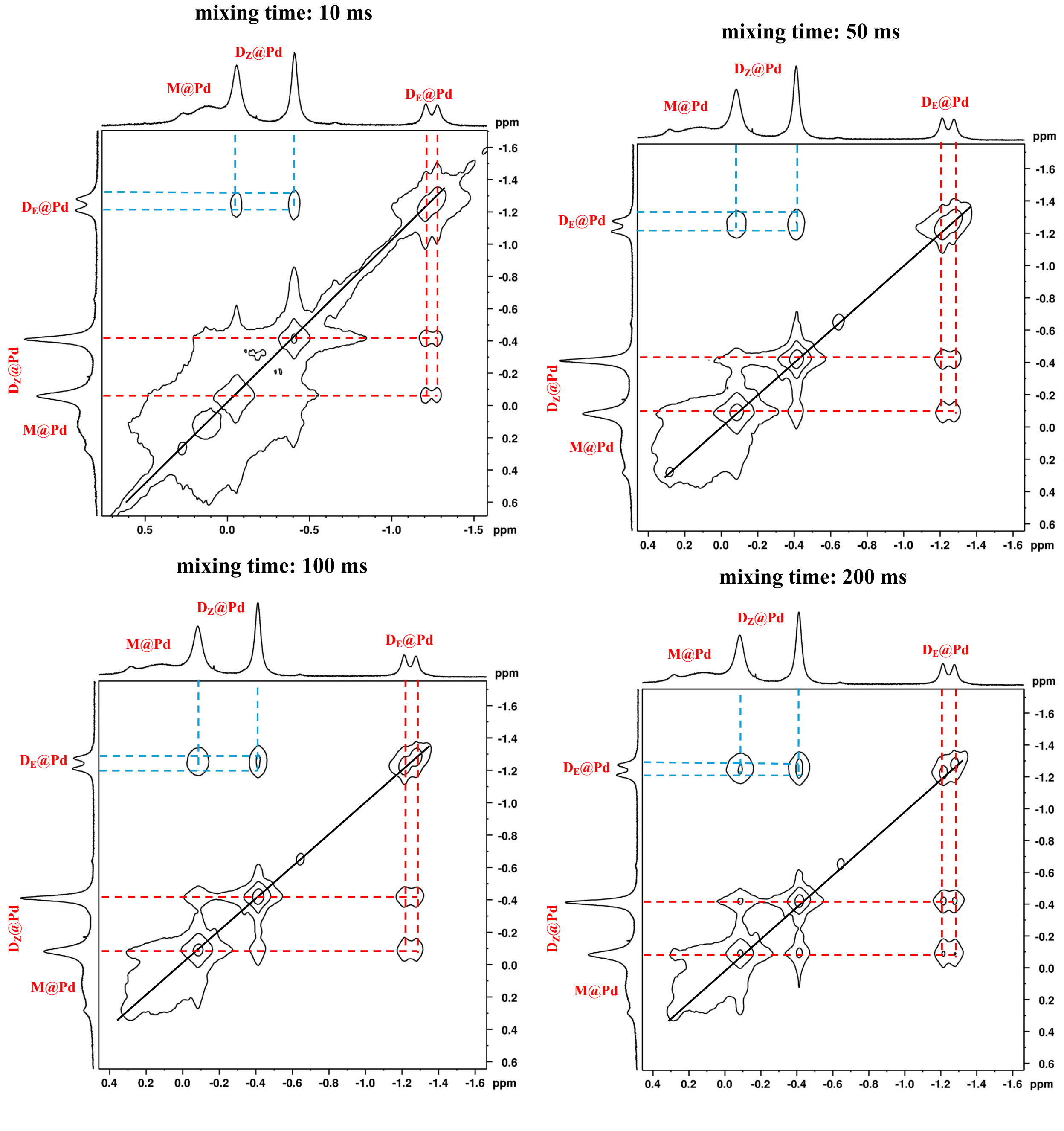

Figure S24: 2D NOESY (800 MHz, D_2O, 5 °C) spectra of 2.0 mM *o*-NC + 0.7 mM PdNC showing exchange correlations between complexed isopropyl groups at 10 - 200 ms mixing times. Red dotted lines indicate exchange correlations between complexed monomers and dimers, while blue dotted lines indicate exchange between D_z and D_E.

Plot of 2D integral value vs exchange buildup obtained at different mixing times

Mixing time (ms)	NOE exchange integrals of D_Z-D_E
20	0.09
50	0.21
100	0.31
200	0.36

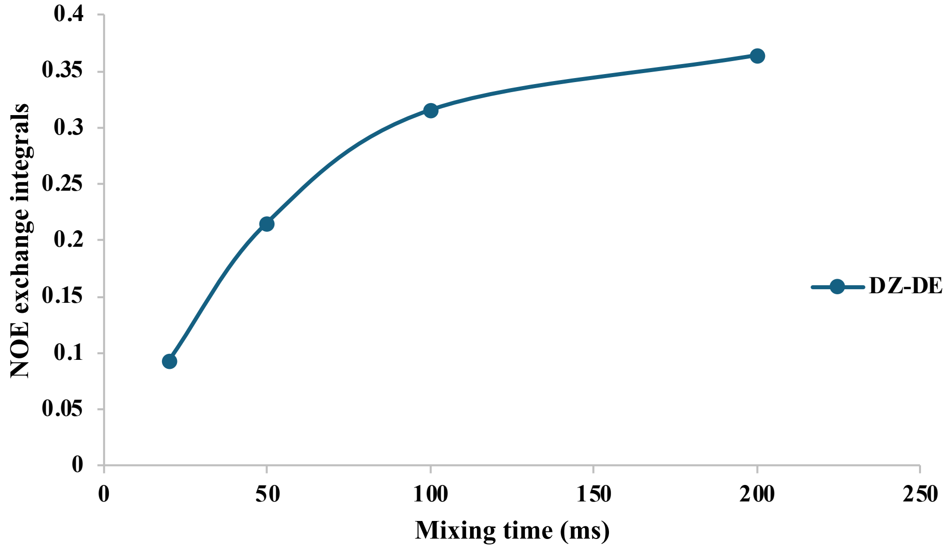

Figure S25: A plot of 2D NOE exchange integrals of D_Z-D_E vs mixing time.

Table S5: Chemical shift differences of isopropyl methyls of both D_Z and D_E dimers upon encapsulation with PdNC.

[*o*-NC] mM	Chemical shift difference of *i*-Pr methyls in ppm	
	D_Z	D_E
**2.5**	0.31	-
**2.2**	0.33	-
**2.0**	0.33	-
**1.5**	0.29	0.08
**1.2**	0.26	0.08
**1.0**	0.24	0.08
**0.9**	0.23	0.08
**0.8**	0.21	0.08
**0.7**	0.20	0.08
**0.6**	0.19	0.08
**0.5**	0.18	0.08
**0.4**	0.16	0.08
**0.3**	0.15	0.09
**0.2**	0.13	0.09
**0.1**	0.10	0.09

B3LYP-631G optimized structures of dimers D_Z and D_E & Rotational video of D_Z

Computational Details

Density functional theory calculations (DFT) were performed using Spartan'20[a, b] with the B3LYP functional using a 6-31G* basis set. Geometry optimization was performed and optimized structures were confirmed to be stationary points by frequency analysis (zero imaginary frequencies for the energy minima, one imaginary frequency for transition states). No solvation correction was applied. For D_E, the calculated cumyl/azodioxide plane/plane twist angle (CCNO) was 58 deg, and for D_Z, this same twist angle (CCNO) was 63 deg (compare to 74 deg observed from D_Z crystal structure[c]).

(a) Deppmeier, B. J.; Driessen, A. J.; Hehre, W. J.; Hehre, T. S.; Johnson, J. A.; Ohlinger, W. S.; Klunzinger, P.E., Spartan'20, Wavefunction Inc., Irvine, CA, **2022**.

(b) Y.Shao *et al.*, Advances in molecular quantum chemistry contained in the Q-Chem 4 program package. *Mol. Phys.* **2015,** 113, 184-215.

(c) Rogers, C. H.; Pradeep, A.; Galiano, L. A.; Kelley, S. A.; Varadharajan, R.; Belmore, K.; Whitt, L. M.; Li, Y.; Champagne, P. A.; Ramamurthy, V.; Blackstock, S. C., Dynamic Covalent and Noncovalent Assembly of *o*-Nitrosocumene in Organic Solvents and Water. *Chem. Commun.* **2024**, *60*, 13899–13902.

(a) Two views of D_E anti structure, E = -958.972821 au (vacuum)

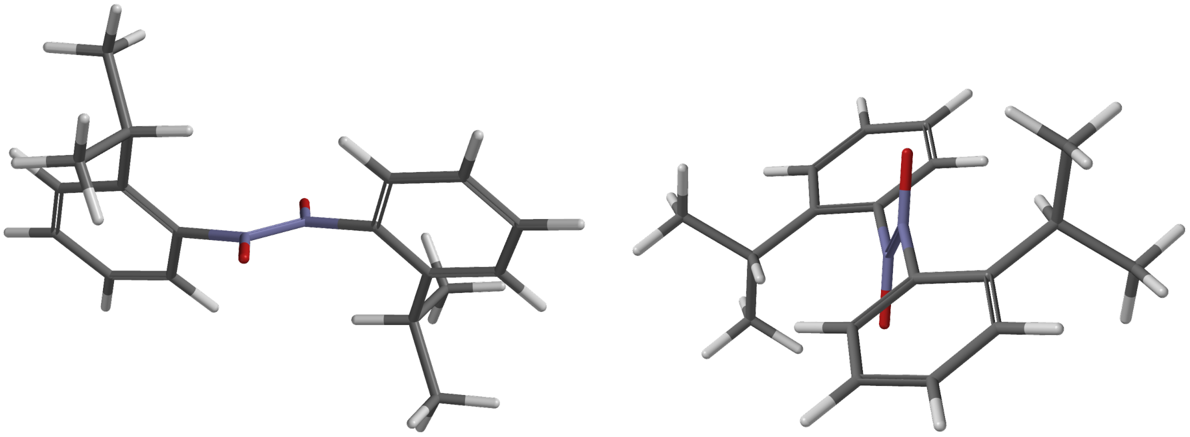

(b) Two views of D_Z anti structure, E = -958.969076 au (vacuum)

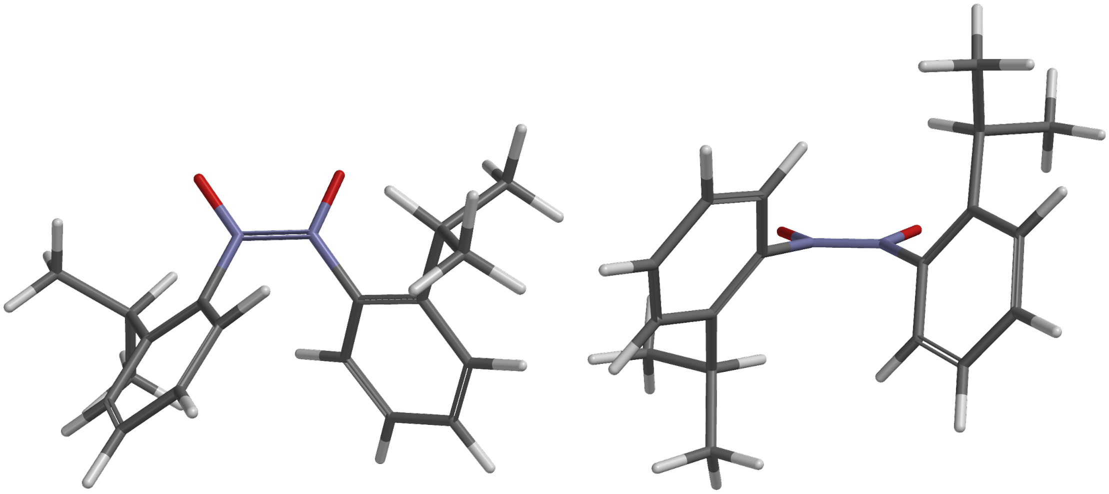

(c) The X-ray crystal structure of D_Z with rotation

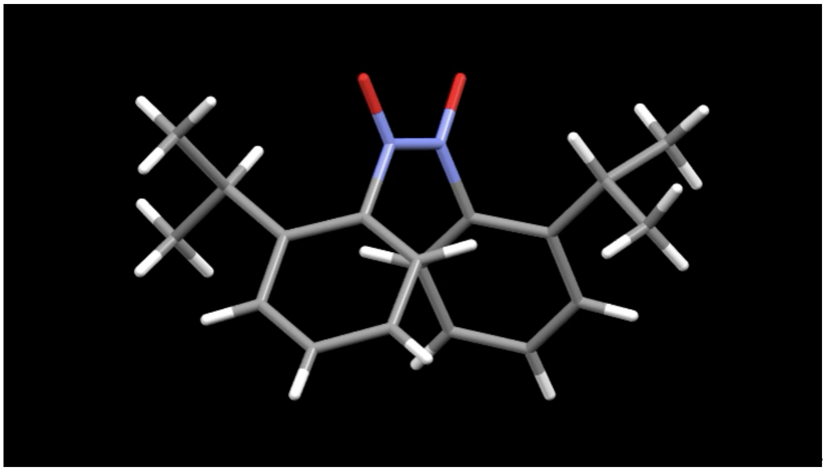

Figure S26: B3LYP-631G optimized structures of dimers (a) D_Z and (b) D_E, (c) D_Z crystal structure showing the twisted cumyl groups with video option. Crystallographic data can be accessed by CCDC 2313924.

XYZ coordinates of all DFT-optimized geometries

***o*-NC** D_E anti structure

Standard Nuclear Orientation (Angstroms)

I	Atom	X	Y	Z
1	H	-1.6202120517	2.1171760948	0.2387720417
2	C	-0.9199830219	2.2697371129	1.0517100656
3	C	0.9228061298	2.6175641268	3.1024772156

4	C	0.0078890279	1.2732890314	1.3603020761
5	C	-0.9265520171	3.4494352144	1.7864401316
6	C	0.0000710685	3.6205912049	2.8172111812
7	C	0.9560970963	1.4118500823	2.3846591794
8	H	-1.6472700505	4.2280372525	1.5550921297
9	H	0.0051860433	4.5384412719	3.3987362202
10	H	1.6390511864	2.7686891694	3.9046262671
11	N	-0.0875529643	0.0010919573	0.6580650302
12	N	0.0787840733	0.0040559322	-0.6566520210
13	C	-0.0114409717	-1.2688501336	-1.3584881093
14	C	0.0087920690	-3.6174252959	-2.8133561775
15	C	-0.9592090291	-1.4134511611	-2.3825371785
16	C	0.9216341079	-2.2602192245	-1.0492460574
17	C	0.9346260983	-3.4405152962	-1.7827901313
18	C	-0.9193150414	-2.6195592309	-3.0993762366
19	H	1.6210241800	-2.1031181827	-0.2364700147
20	H	1.6598641524	-4.2147533680	-1.5509300975
21	H	-1.6348080842	-2.7755022212	-3.9012492693
22	H	0.0086060631	-4.5356603923	-3.3942942531
23	O	-0.3231659763	-1.0648170991	1.3083151173
24	O	0.3138570599	1.0701450147	-1.3068721037
25	C	1.9743931617	0.3316919749	2.7298071803
26	H	1.9431521751	-0.4275880542	1.9433851190
27	C	1.5915621817	-0.3767340639	4.0437313123
28	H	2.3076162053	-1.1760831326	4.2690033006
29	H	0.5957250882	-0.8206690924	3.9597932638
30	H	1.5911231762	0.3224369817	4.8887193290
31	C	3.4097722654	0.8858339944	2.7813592083
32	H	3.6794983209	1.3853980444	1.8444031424
33	H	4.1205403371	0.0683479563	2.9485092208
34	H	3.5434153014	1.6060970942	3.5967762690
35	C	-1.9811871101	-0.3377430643	-2.7303082053
36	H	-1.9602680970	0.4178339938	-1.9398611157
37	C	-3.4132972212	-0.8984981065	-2.7955351798
38	H	-4.1265772446	-0.0831200461	-2.9620902384
39	H	-3.6870102042	-1.4054571445	-1.8637381162
40	H	-3.5384691871	-1.6138251464	-3.6167112770
41	C	-1.5911200541	0.3790509608	-4.0376293161
42	H	-0.5978079829	0.8263339882	-3.9432353066
43	H	-2.3086801163	1.1765910369	-4.2646502882
44	H	-1.5811600908	-0.3161300880	-4.8858733606

***o*-NC** D_Z anti DFT-optimized geometry

Standard Nuclear Orientation (Angstroms)

I	Atom	X	Y	Z
1	H	-1.5263331500	1.1532261155	-1.0776461343
2	C	-1.4568300314	1.2657914550	-0.0007099680
3	C	-1.3326692345	1.4830589270	2.7670146459
4	C	-1.2028893113	0.1476580713	0.7954787478
5	C	-1.6353422912	2.5092859189	0.5986067501
6	C	-1.5704989920	2.6148258052	1.9883611297
7	C	-1.1456439704	0.2160846865	2.1975826830
8	H	-1.8321339428	3.3834748693	-0.0143272665
9	H	-1.7129159506	3.5787984080	2.4692239105
10	H	-1.2933730474	1.5836895059	3.8475164029
11	N	-1.1204993453	-1.1538728768	0.1563899049
12	N	-0.1329120155	-1.4548653945	-0.6883267737
13	C	0.9953969663	-0.5612630979	-0.8824755495
14	C	3.2640100030	0.9738171239	-1.2569493498
15	C	1.8687348614	-0.3525421011	0.1858917266
16	C	1.2308510264	-0.0358934014	-2.1640664501
17	C	2.3887004798	0.7393223130	-2.3163871074
18	C	3.0065074441	0.4270394994	0.0003884712
19	H	1.6580969132	-0.8085761802	1.1474275023
20	H	2.6121250782	1.1664595555	-3.2895940498
21	H	3.6888245865	0.5961900532	0.8277360595
22	H	4.1519775890	1.5793710662	-1.4163793094
23	O	-0.1670443598	-2.5551831944	-1.3029476969
24	O	-2.0192235395	-2.0092315805	0.3768226082
25	C	0.3011267992	-0.3069119269	-3.3410412442
26	H	-0.6248395558	-0.7370964769	-2.9475207287
27	C	-0.0628833262	0.9764142940	-4.1080859892
28	H	0.8066959891	1.4139743607	-4.6118284303
29	H	-0.4831616543	1.7422183751	-3.4448457613
30	H	-0.8078235307	0.7515901420	-4.8793598119
31	C	0.9147715756	-1.3666330188	-4.2769787913
32	H	1.8648402958	-1.0191038387	-4.7015405313
33	H	0.2320320977	-1.5821297948	-5.1073144849
34	H	1.0949868059	-2.2956713385	-3.7296680817
35	C	-0.9207163544	-1.0135089955	3.0694071865
36	H	-0.5954447256	-1.8329467426	2.4216529512
37	C	-2.2452876473	-1.4637311894	3.7167228887
38	H	-2.6486634362	-0.6866107474	4.3771418154
39	H	-2.0856653369	-2.3672988523	4.3164688201
40	H	-2.9859220291	-1.6877473132	2.9440152192
41	C	0.1722202199	-0.7899204620	4.1301567332

42	H	-0.1245152868	-0.0410338031	4.8750966138
43	H	1.1141743472	-0.4564265603	3.6799073629
44	H	0.3671589871	-1.7240916583	4.6689833758
